# Leukodystrophies: a proposed classification system based on pathological changes and pathogenetic mechanisms

**DOI:** 10.1007/s00401-017-1739-1

**Published:** 2017-06-21

**Authors:** Marjo S. van der Knaap, Marianna Bugiani

**Affiliations:** 1 Department of Pediatrics/Child Neurology, VU University Medical Centre, Amsterdam Neuroscience, Amsterdam, The Netherlands; 2 Department of Functional Genomics, Centre for Neurogenomics and Cognitive Research, Amsterdam Neuroscience, VU University, Amsterdam, The Netherlands; 3 Department of Pathology, VU University Medical Centre, Amsterdam Neuroscience, Amsterdam, The Netherlands

**Keywords:** Leukodystrophy, Myelin, Astrocytes, Oligodendrocytes, Microglia, Axons

## Abstract

Leukodystrophies are genetically determined disorders characterized by the selective involvement of the central nervous system white matter. Onset may be at any age, from prenatal life to senescence. Many leukodystrophies are degenerative in nature, but some only impair white matter function. The clinical course is mostly progressive, but may also be static or even improving with time. Progressive leukodystrophies are often fatal, and no curative treatment is known. The last decade has witnessed a tremendous increase in the number of defined leukodystrophies also owing to a diagnostic approach combining magnetic resonance imaging pattern recognition and next generation sequencing. Knowledge on white matter physiology and pathology has also dramatically built up. This led to the recognition that only few leukodystrophies are due to mutations in myelin- or oligodendrocyte-specific genes, and many are rather caused by defects in other white matter structural components, including astrocytes, microglia, axons and blood vessels. We here propose a novel classification of leukodystrophies that takes into account the primary involvement of any white matter component. Categories in this classification are the myelin disorders due to a primary defect in oligodendrocytes or myelin (hypomyelinating and demyelinating leukodystrophies, leukodystrophies with myelin vacuolization); astrocytopathies; leuko-axonopathies; microgliopathies; and leuko-vasculopathies. Following this classification, we illustrate the neuropathology and disease mechanisms of some leukodystrophies taken as example for each category. Some leukodystrophies fall into more than one category. Given the complex molecular and cellular interplay underlying white matter pathology, recognition of the cellular pathology behind a disease becomes crucial in addressing possible treatment strategies.

## Introduction: what is a leukodystrophy?

Leukodystrophies are heritable, mostly progressive encephalopathies characterized by the selective involvement of the central nervous system (CNS) white matter. The first report of a familial white matter disorder dates back over a century, when Pelizaeus and Merzbacher separately described the familial occurrence of a chronic progressive ‘diffuse sclerosis’ (as opposed to the already recognized ‘multiple sclerosis’) with lack of myelin and sclerotic hardening of the white matter [138, 169]. The term “leukodystrophy” (*leuko*, white and *dystrophy*, wasting) was used for the first time in 1928 in the context of metachromatic leukodystrophy and coined to define hereditary, progressive diseases characterized by white matter degeneration [16]. In the 1980s [145], leukodystrophies were considered genetic, progressive disorders primarily affecting myelin, either directly or through oligodendrocytes. At that time, the diseases were pathogenetically poorly characterized with an unknown molecular basis; data were available from pathology, biochemical analyses of brain tissue and knowledge of some metabolic and enzymatic defects, but no gene defects. Soon after, MRI came into use as primary tool to diagnose leukodystrophies, while no pathological data were available to confirm the primary myelin involvement. In the last two decades many gene defects have been identified, first by genetic linkage and more recently by whole exome and genome sequencing. Because many of these disorders prove to be caused by defects in housekeeping processes, the myelin-focused definition of term leukodystrophy has been recently considered too narrow [103].

How should we define leukodystrophies at this time? The term leukodystrophy in its intentional meaning is not applicable to all genetic white matter disorders, because many are not progressive or characterized by primary myelin loss. This term has survived because of its popularity, but has lost its precision in the light of the current knowledge. Many use the term “leukoencephalopathy” to define all disorders that affect exclusively or predominantly the brain white matter [102]. Although linguistically correct, this choice does not distinguish genetic from acquired disorders, degenerative from non-degenerative diseases, and progressive from static conditions. Leukodystrophies were recently redefined as “heritable disorders affecting the white matter of the central nervous system, sharing glial cell or myelin sheath abnormalities, the neuropathology of which is primarily characterized by involvement of oligodendrocytes, astrocytes and other non-neuronal cell types, although in many disorders the mechanism of disease remains unknown, and in other cases is suspected to include significant axonal pathology” [259]. With this, the word leukodystrophy has become a term to indicate all inherited white matter disorders [103]. Some may consider this choice to be infelicitous as well, because the term would then define both degenerative disorders, as in its original and still widely perceived meaning, and static, episodic or even improving conditions [130, 208]. Obviously, there is no perfect definition of the word leukodystrophy. It is colored by the state of knowledge at the time of the definition and therefore subject of change. Leukodystrophies are currently defined as all genetically determined disorders primarily affecting central nervous system white matter, irrespective of the structural white matter component involved, the molecular process affected and the disease course [103].

## White matter integrity and function: teamwork is required

The white matter comprises half of the human brain. It has expanded more than gray matter during evolution [274], and constitutes an indispensable component of the neural networks that subserve motor and cognitive operations. White matter tracts mediate the essential connectivity by which brain function is organized, working in concert with gray matter to enable the extraordinary repertoire of human neurobehavioral capacities [60].

The white matter is composed of myelinated axons, glial cells (myelinating oligodendrocytes and oligodendrocyte progenitor cells [OPCs], NG2-glia, astrocytes and microglia) and blood vessels, all embedded in the extracellular matrix (ECM). CNS white matter is half myelin and half non-myelin on a dry weight basis. The myelin sheath is an extended and modified plasma membrane wrapped around the axons that originates from and is part of oligodendrocytes [67]. Myelin acts as a high resistance, low capacitance electrical insulator that facilitates conduction while preserving space and energy [149]. Myelin also supports the long-term structural integrity and viability of axons [148, 149] and provides essential trophic support by delivering glycolysis products for mitochondria in long fiber tracts [64, 148, 149]. The generation of myelin is tightly regulated by the interplay of intrinsic oligodendrocytic cues and extrinsic cues originating from neighboring glial and not-glial white matter cells and ECM components [53, 144]. It involves partly overlapping steps of OPC specification, proliferation, migration and morphological differentiation culminating in the generation of compact myelin around appropriate receptive axons. Many of these regulatory mechanisms are also essential after development for white matter maintenance and repair [55].

### Oligodendrocytes and myelin

OPCs are identified by their concurrent expression of the pan-lineage marker Olig2, the chondroitin sulfate proteoglycan NG2 and the platelet-derived growth factor receptor alpha (PDGFRα). During development, they are generated in distinct waves through time and space. The brain produces an overabundance of OPCs, but a large percentage of these cells die as they compete for limited astrocytic and axonal factors [12, 13, 229]. A substantial number of OPCs persist in the adult brain, where they actively proliferate and are involved in myelin remodeling, de novo myelination of unmyelinated axons and remyelination upon injury [273].

Regulation of OPC migration ensures that adequate numbers of OPC reach the final site of myelination, through signals provided by white matter cells other than oligodendrocytes. Extracellular effectors regulating OPC migration include motogenic factors stimulating OPC motility, adhesion and contact molecules present in the ECM, and long-distance chemotactic cues [53, 144, 156]. Notably, axonal signals also regulate OPC proliferation and migration. Neuregulin-1 (NRG-1), for example, acts as proliferation signal as well as a differentiation cue [40].

Once they have reached their final destination, OPCs terminally differentiate into myelin-forming oligodendrocytes. This is a key point in the myelination process. In mice, OPC terminal differentiation and myelination are almost concurring events, with pre-myelinating cells rapidly progressing to myelination or undergoing apoptosis [12, 229]. By contrast, the developing human brain appears to harbor pre-myelinating oligodendrocytes for longer periods, before these cells finally start to myelinate [7]. Greater complexity of the human brain, including its larger size and longer development, and the existence of unique regions and functions, presumably account for the need of a greater potential and more complex regulation of oligodendrocyte differentiation and myelination. The balance between OPC proliferation and terminal differentiation is tightly regulated to ensure that oligodendrocyte lineage progression takes place in an orderly sequence and prevent differentiated patterns of gene expression from being induced prematurely or in the wrong cells [93]. Indeed, many of the factors participating in this process are inhibitory and of axonal and astrocytic origin [8, 38, 99, 127, 191]. Interestingly, there is evidence that the level of axonal activity also impacts OPC terminal differentiation. Release of adenosine by active axons activates purinergic receptors on OPCs and promotes differentiation, and axonal release of ATP stimulates adjacent astrocytes to secrete pro-myelination factors [97].

The final stage of oligodendrocyte development is myelination. Myelination occurs in a very short time window in the lifetime of the individual oligodendrocyte, during which myelin sheaths are formed and the number of sheaths is determined [42]. For this to take place, intrinsic and extrinsic regulators interact dynamically to control the balance between differentiation and myelination in a spatiotemporally specific manner. Many extrinsic ligands influencing myelination are axonal. They act by preventing myelination initiation and excessive myelination [59, 140] or by promoting myelination via reorganization of the oligodendrocyte cytoskeleton and the extension and branching of its processes [14, 123, 180]. Axonal signals are also required to establish adequate myelin thickness, possibly as a reflection of neuronal activity [132, 220, 275].

### Astrocytes

Astrocytes are a highly prevalent cell population in the brain. They are an extremely heterogeneous cell type essential for brain development and maintenance of CNS homeostasis [205]. Astrocytes induce and preserve the integrity of the blood–brain and blood–cerebrospinal fluid barriers, control the extracellular ionic milieu, provide metabolic support to neurons, facilitate perivascular flow of cerebrospinal fluid, ensure proper synaptic transmission and plasticity, and are involved in cerebral blood flow regulation [11, 96, 205]. They also participate in regulating developmental myelination and myelin maintenance in the adult brain [9]. In vitro and in vivo studies have shown that astrocytes are a major source of many regulatory signals that influence OPC survival, oligodendrocyte differentiation, maturation and myelination. Astrocytes also secrete ECM components and are involved in ECM remodeling, which may affect OPC proliferation, differentiation and myelination [9, 87]. The impact of astrocytes on white matter function and integrity was definitively confirmed by the identification of human white matter disorders linked to mutations in astrocyte-specific gene products such as the intermediate filament glial fibrillary acidic protein (GFAP, Alexander disease) [25] and MLC1 (megalencephalic leukoencephalopathy with subcortical cysts, MLC) [125].

Astrocytes contribute to maintenance of white matter integrity and function also by orchestrating the control of ion–water homeostasis and preventing intramyelinic edema [15, 181]. When action potentials are transmitted through the white matter, depolarization of myelinated axons is associated with influx of sodium at the nodes of Ranvier and compensatory efflux of potassium at the paranodal regions covered by myelin. These fluctuations of ions are accompanied by osmotically driven shifts in water that require immediate compensation to allow further impulse transmission and prevent cellular swelling and intramyelinic edema. Excessive osmotic water and potassium are siphoned away across the paranodal myelin into astrocytes. Long-distance disposal of water and ions occurs via dispersion through the panglial syncytium, a network of astrocytes, oligodendrocytes and ependymal cells also interconnected by gap junctions. The crucial role of astrocytes in maintaining myelin integrity by potassium siphoning and gap junction communication is shown by extensive white matter vacuolization in mice lacking gap junctions that form heterotopic interactions between oligodendrocytes and astrocytes [133, 230] and human white matter disorders due to defects in astrocytic proteins crucial for ion–water homeostasis [45, 50].

### Axons

Axons and the ensheathing glia interact bidirectionally and throughout life. This interaction is essential for both partners: lack of myelin leads to axonal degeneration and axonal degeneration leads to loss of myelin [20, 52]. As mentioned above, axonal signals participate in regulating oligodendroglial lineage progression and myelination in vivo [276]. Axonal ligands also control myelination initiation, mediate the influence on myelination of ECM components [59, 180], regulate membrane trafficking in oligodendrocytes [106, 228] and are required to establish adequate myelin thickness [124, 220].

In addition, neuronal activity directly influences myelination [44]. Blockade of activity in the developing rat optic nerve decreases OPC proliferation [13] whereas increased electrical activity enhances OPC proliferation and differentiation or the rate of myelin development [68, 126]. This puts forward the intriguing possibility that abnormal neuronal activity in genetic diseases affecting the human cortex may as well impact on white matter integrity.

As a consequence of the close interaction between axons and the ensheathing glia, myelination is perturbed when axonal dysfunction and degeneration starts before myelination has reached completion, as it happens in infantile-onset lysosomal neuronal storage disorders as gangliosidoses and neuronal ceroid lipofuscinoses (NCL), and in the white matter underlying the dysplastic cortex in Zellweger syndrome. *Cln8*-deficient mice modeling NCL show delayed myelination and increased OPC numbers, suggesting a defect in OPC maturation [115]. Neuropathology of patients with GM1 and GM2 gangliosidoses reveals failed myelin development and paucity of oligodendrocyte lineage cells, which may be compatible with a defect in OPC proliferation or survival [75, 250].

### Microglia

Microglia are the main innate immune cells of the CNS. In contrast to oligodendrocytes and astrocytes that originate from neural progenitors within the neuroectoderm, microglia arise from hematopoietic stem cells in the yolk sac during embryogenesis and migrate to populate the CNS [70]. Microglia are critically involved in maintaining homeostasis during and after development. Being the major immune effectors of the CNS, they also act as surveillance cells and sensors of pathologic events [85]. In the white matter, microglia contribute to regulation of myelin maintenance and play a role upon injury and during repair. In homeostatic conditions, microglia promote OPC survival and differentiation and myelination [81, 92, 152, 166]. Upon injury, microglia play dual roles also depending on their polarization status, either hindering OPC differentiation [161] and inducing oligodendrocyte apoptosis [271] or promoting OPC differentiation and remyelination [114, 142]. Another important aspect of microglia concerns its role in the clearance of myelin debris in the case of white matter damage with myelin loss [117, 151, 204]. This step is crucial in the remyelination process and underscores the importance of microglia during white matter repair. The impact of microglia on white matter function and integrity was confirmed by the identification of human white matter disorders linked to mutations in microglia-specific gene products, including hereditary diffuse leukoencephalopathy with axonal spheroids (HDLS) and pigmented orthochromatic leukodystrophy (POLD) [109], due to changes in the colony stimulating factor 1 receptor (CSF1R) involved in microglia homeostasis, and Nasu Hakola disease linked to changes in the tyrosine kinase binding adaptor protein and the triggering receptor expressed on myeloid cells 2 (TYROBP and TREM2, respectively), that play a role in the phagocytic activity of microglia.

## A novel classification of genetic white matter disorders based on a cellular pathology approach

Every classification reflects the knowledge of its time. The current classification of white matter disorders recognizes four categories: hypomyelinating (i.e., lack of myelin deposition), demyelinating (i.e., loss of previously deposited myelin), dysmyelinating (i.e., deposition of structurally or biochemically abnormal myelin) and myelinolytic diseases [147] (i.e. myelin vacuolization). This classification has the major value of categorizing white matter disorders according to main mechanism of white matter injury and recognizing the possibility that different pathomechanisms may contribute to a single disease. One could, however, question the choice of terms arguing that, also in the light of more recent insights on white matter integrity and function, their reflection of the different disease categories is no longer tenable and that more pathomechanisms may play a primary role in white matter pathology than those four alone.

We therefore put here forward a new classification of genetic white matter disorders that better reflects the scientific knowledge of this time (Table 1). The contribution of cell types other than oligodendrocytes and structures other than myelin driving white matter pathology, including astrocytes, neurons, microglia and blood vessels, is considered to provide additional information as to the pathogenesis. Importantly, given the complex mechanisms underlying many white matter disorders, the classification recognizes the possibility that a specific disease does not primarily affect one cell type or structure only and with that belongs to more than one category.

**Table 1:** A new classification of genetic white matter disorders

Myelin disorders	Leuko-axonopathies
Hypomyelination	a. Hypomyelination with atrophy of the basal ganglia and cerebellum [80]
a. Pelizaeus-Merzbacher disease [224]	b. Hypomyelination with congenital cataract [66]
b. Peripheral neuropathy, central hypomyelination, Waardenburg-Hirschsprung [36]	c. Early-onset neuronal degenerative disorders
c. Cx47-related Pelizaeus-Merzbacher-like disease [36]	1. Gangliosidosis GM1 and GM2 [75, 250]
d. Hypomyelination of early myelinated structures [104]	2. Infantile neuronal ceroid lipofuscinosis [79]
Demyelination	3. *AGC1*-related disease [265, 268]
a. Metachromatic leukodystrophy [214]	4. *AIMP1*-related diseases [58]
b. Multiple sulfatase deficiency [214]	5. *HSPD1*-related disease [134]
c. Globoid cell leukodystrophy (Krabbe disease) [214]	d. Pol III-related leukodystrophies [269]
d. X-linked adrenoleukodystrophy, cerebral from [173]	e. Leukoencephalopathy with brainstem and spinal cord involvement and high lactate [231]
Myelin vacuolization	f. Hypomyelination with brainstem and spinal cord involvement and leg spasticity [216]
a. Mitochondrial diseases with leukoencephalopathy [159]	g. Giant axonal neuropathy [135]
b. Phenylketonuria [94]	Microgliopathies
c. Canavan disease [91]	a. *CSF1R*-related disorders [153, 179]
d. Other selected disorders of amino acid metabolism [2]	1. Hereditary diffuse leukoencephalopathy with spheroids
e. Cx32-related (X-linked) Charcot-Marie-Tooth disease [45]	2. Pigmentary ortochromatic leukodystrophy
Astrocytopathies	b. Nasu-Hakola disease [193]
a. Alexander disease [25]	Leuko-vasculopathies
b. Megalencephalic leukoencephalopathy with subcortical cysts [23]	a. Cerebral AD arteriopathy with subcortical infarcts and leukoencephalopathy [162]
c. ClC-2-related disease [45]	b. Cerebral AR arteriopathy with subcortical infarcts and leukoencephalopathy [162]
d. Vanishing white matter [48]	c. Cathepsin A-related arteriopathy with strokes and leukoencephalopathy [31]
e. Aicardi-Goutières syndrome and variants [255]	d. Cerebral amyloid angiopathy [162]
f. Oculodentodigital dysplasia (Cx43) [1]	e. Leukoencephalopathy with calcifications and cysts [98]
g. Giant axonal neuropathy [135]	

The table is meant to give examples and not to be exhaustive

*AD* autosomal dominant, *AR* autosomal recessive

We propose to classify white matter disorders into six main categories:

- A first category of “myelin disorders” includes those disorders in which oligodendrocytes and myelin are primarily or predominantly affected. These are the hypomyelinating disorders, the demyelinating disorders, and the diseases with myelin vacuolization.
- A second category comprises white matter disorders due to defects in astrocyte-specific gene products or in which astrocyte dysfunctions play a major pathogenetic role: the “astrocytopathies”.
- A third category encompasses white matter disorders secondary to neuronal or axonal defects. We adopt the term “leuko-axonopathies” for this category, to highlight that the white matter degeneration results from an abnormal axo-glia interaction.
- A fourth category comprises white matter disorders due to defects in microglia-specific gene products: the “microgliopathies”.
- A fifth category contains genetic white matter disorders due to vascular pathology: the “leuko-vasculopathies”.

Not all white matter disorders that can be currently diagnosed have been pathologically characterized. For this reason, the assignment of a certain condition to one or the other category also depends on data derived from imaging studies and, when known, on the supposed function of the associated mutated protein. For some white matter disorders, the cellular pathomechanisms are presently still so unclear that proper classification is not possible.

## Pathology and mechanisms of genetic white matter disorders: some examples

### Myelin disorders

Myelin disorders comprise diseases in which myelin deposition is permanently deficient (hypomyelination), in which myelin is first normally deposited and later lost (demyelination) and those in which myelin integrity is disrupted because of primary or secondary intramyelinic vacuolization. The common neuropathological and pathogenetic denominator of myelin disorders is the primary or predominant involvement of oligodendrocytes and/or myelin.

#### Myelin disorders with hypomyelination: Pelizaeus-Merzbacher disease

Hypomyelinating diseases are a group of neurodevelopmental disorders that affect the proper formation of the myelin sheath in the CNS. As a group, they are clinically characterized by developmental delay, hypotonia, ataxia, spasticity, and variable intellectual disability. This group includes Pelizaeus–Merzbacher disease (PMD), caused by *PLP1* gene mutations, and numerous other disorders assigned to defects in *GJC2*, *AIMP1*, *HSPD1*, *FAM126A*, *POLR3A*, *POLR3B*, *RARS*, *PYCR2*, *POLR1C*, and *VPS11* [36].

The prototype hypomyelinating disorder PMD is an X-linked condition caused by changes in *PLP1* encoding proteolipid protein 1 (PLP1) and its alternatively spliced form DM20. The PLP1/DM20 protein is one of the main structural components of the myelin sheath [110]. *PLP1* changes give rise to a spectrum of disorders with a strict genotype–phenotype correlation. The most common variants, *PLP1* duplications, cause the classical form of PMD. Missense mutations give rise to a clinically more severe form of PMD with connatal onset, while deletions and null mutations give rise to null PMD syndrome and spastic paraplegia type 2 [90].

PMD is characterized by onset in the first months of life of nystagmus, developmental delay, hypotonia, ataxia and spasticity, feeding and breathing issues, involuntary movements and epilepsy. MRI shows diffuse hypomyelination, i.e., homogeneous white matter mild hypo- or isointensity relative to gray matter structures on T1-weighted images and mild hyperintensity on T2-weighted images, and ensuing white matter atrophy over time.

On macroscopic examination PMD brains are small and, on sectioning, show dilation of the lateral ventricles and thinning of the corpus callosum. The white matter of the centrum semiovale, cerebellum, brainstem and spinal cord appears shrunken and gray with a variably gelatinous or firm consistency. The optic nerves are thin and gray, in sharp contrast to the other cranial nerves and spinal nerve roots that have a normal size and are white. Histopathology may vary according to the type of *PLP1* mutation [122]. In classical PMD due to *PLP1* gene duplications, microscopic analysis shows paucity of myelin with a classic tigroid distribution due to preservation of myelin islets around blood vessels. Oligodendrocytes are markedly reduced in numbers to absent, especially if myelin is completely lacking. There is astrocytosis, fibrillary gliosis and robust microglia cell activation. Very sparsely, perivascular macrophages contain sudanophilic lipid material.

Lack of myelin is thought to be a consequence of oligodendrocyte death. *PLP1* point mutations and duplications confer a toxic gain of function in oligodendrocytes, with consequent misfolding and aggregation of mutated PLP1 [46]. Under normal conditions, PLP1 is synthesized at the endoplasmic reticulum (ER)/Golgi apparatus, associates with lipids and is transported to myelin by vesicular transport [224]. *PLP1* point mutations prevent normal trafficking of the PLP1 protein to the cytoplasmic membrane and cause its aggregation in the ER and Golgi apparatus with activation of the unfolded protein response. Overdosage of *PLP1* due to gene duplication leads to excessive PLP1 accumulation at the late endosomes and lysosomes with accompanying cholesterol sequestration [201]. Irrespective of the site, mutant PLP1 accumulation induces apoptotic cell death of oligodendrocytes [224]. The greater the accumulation of mutated PLP1 protein, the higher the likelihood of apoptosis and increased disease severity [206]. Additionally, depletion of chaperones in the ER and Golgi fragmentation induced by mutant unfolded proteins contribute to trafficking defects and could contribute to the pathogenesis of PMD [158].

As PLP1 is mainly expressed in oligodendrocytes, cell replacement therapy is a promising approach to treat PMD. A recent clinical trial showed that transplantation of human neural precursor cells (hNPC) in children with PMD is safe, although with minor impact on clinical recovery [74]. The therapeutic benefits of engrafting hNPCs versus human OPCs that are already committed to the oligodenrocyte lineage were investigated in immunodeficient *Plp1*-overexpressing mice [137]. Although both cell types were able to differentiate and restore compact myelin in PMD mice, only transplantation of hNPCs significantly increased the host survival, suggesting that myelin restoration alone is not sufficient to rescue the PMD phenotype. Prolonged survival of hNPC-transplanted mice correlated with reduced astrogliosis and microgliosis, and with a switch of macrophages/microglia polarization from a classically activated M1 proinflammatory phenotype towards an alternatively activated M2-like repair phenotype. This indicates that besides myelin restoration modulation of inflammation may be necessary to promote clinical recovery.

#### Myelin disorders with demyelination: metachromatic leukodystrophy

Metachromatic leukodystrophy (MLD) is an autosomal recessive lysosomal disorder due to *ARSA* gene mutations resulting in deficiency of the enzyme arylsulfatase A (ASA). Low ASA activity causes the accumulation of sulfatides in the central and peripheral nervous system leading to demyelination. MLD is classified in a late-infantile, juvenile and adult-onset type based on the age of the first symptoms, with the disease type correlating to the kind of ARSA mutation and degree of residual ASA activity [69, 257].

**Fig. 1:**
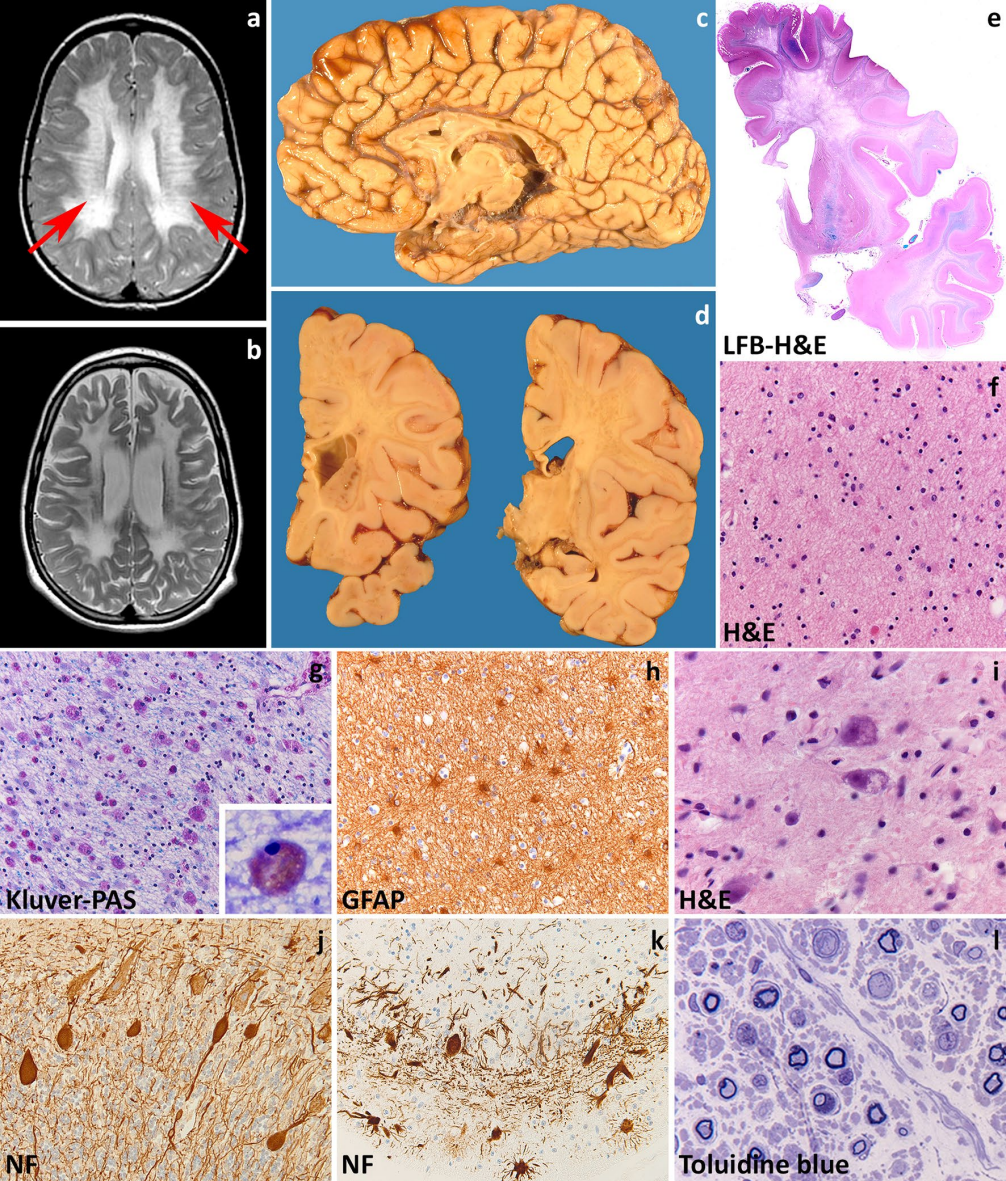
Metachromatic leukodystrophy. **a** T2-weighted axial image of a 7-year-old child shows radiating stripes of tissue with preserved signal (*arrows*). The U-fibers are spared. **b** Follow-up T2-weighted axial image of the same child at 13 years shows a diffuse, bilateral and symmetric signal hyperintensity in the cerebral white matter. The U-fibers are no longer spared and the stripes are less well visible. There is a mild atrophy. **c** Sagittal cut of the brain of a 12-year-old child shows thinning of the corpus callosum and optic nerves. **d** On coronal sections through the brain of a 6-year-old child, the demyelinated white matter appears grayish and gelatinous. **e** Whole mount of a coronal section of a 10-year-old child stained with Luxol fast blue and Haematoxylin & Eosin shows diffuse loss of myelin in the frontal and temporal lobe with relative sparing of the U-fibers and internal capsule. **f** Haematoxylin & Eosin stain of the peripheral cerebral white matter shows tissue pallor, loss of oligodendrocytes and presence of foamy macrophages and reactive astrocytes. **g** Klüver-Periodic acid Schiff (PAS) stain of the same area shows loss of myelin and diffusely distributed macrophages filled with PAS-positive granular material. **h** Stain against the glial fibrillary acidic protein (GFAP) shows a moderate diffuse isomorphic astrogliosis. The inset shows a metachromatic macrophage stained with Toluidine blue. **i** Haematoxylin & Eosin stain of the thalamus shows accumulation of storage material in the cytoplasm of neurons. **j**, **k** Stain against neurofilaments (NF) shows axonal swellings (**j**) and dendritic varicosities (**k**) in the cerebellar cortical Purkinje cells. Note also the marked dropout of granular neurons in (**k**). **l** Toluidine blue stain of a semithin section of the sural nerve shows demyelination with accruing of foamy macrophages

In the late-infantile form, signs appear before 30 months with psychomotor regression, irritability, ataxia, peripheral neuropathy, dysphagia and seizures. Death occurs within a few years after the onset. The juvenile variant has its onset between 30 months and 16 years of age. It often features cognitive deterioration and behavioral changes, later followed by central and peripheral motor deterioration and epilepsy. The disease duration is variable. The adult variant has an insidious onset after 16 years with cognitive and behavioral changes and later polyneuropathy. Disease progression is generally slower with death occurring after decades. MRI shows bilateral symmetric hyperintensities on T2-weighted images starting in the corpus callosum, progressing to the periventricular white matter and initially sparing the subcortical fibers. Typical for MLD is a pattern of radiating stripes with normal signal intensity within the abnormal white matter (Fig. 1a, b) [252]. In more severe cases, involvement of the cerebellar white matter, basal nuclei and thalami can also occur. Accumulation of sulfatides also occurs in visceral organs, most often the gallbladder [258].

The degree of neuropathological changes in MLD depends on disease onset [214]. Macroscopically, the brain appears normal to variably atrophic, with atrophy also involving the cerebellum, brainstem and optic nerves (Fig. 1c). On sectioning, the demyelinated white matter is firm to the touch and slightly grayish with relative preservation of myelin in the U-fibers (Fig. 1d, e). These changes are marked in the late-infantile form, mild in the juvenile variant and may not be appreciable in adult-onset patients. Microscopy is characteristic with demyelination accompanied by numerous diffusely scattered macrophages containing hypereosinophilic, PAS-positive globular deposits that are typically metachromatic in frozen sections stained with toluidine blue or acidic cresyl violet (Fig. 1f, g). Increasing demyelination is associated with reduction of oligodendrocyte numbers and increasing reactive gliosis (Fig. 1h). The stripes seen on MRI are related to perivascular preservation of myelin [252]. Metachromatic deposits correspond to lysosomal accumulation of sulfatides that are also present in glial cells and neurons. Neuronal sulfatides storage is mostly appreciated in the spinal gray matter, brainstem cranial nerve nuclei, dentate nucleus in the cerebellum, thalamus, globus pallidus, and retinal ganglion cells (Fig. 1i). Cerebral cortical neurons and cerebellar Purkinje cells are hardly involved (Fig. 1j, k). In the peripheral nerves, segmental demyelination is seen together with metachromatic deposits in Schwann cells and endoneurial macrophages (Fig. 1l). On electron microscopy, sulfatide inclusions show characteristic herringbone or honeycomb patterns. Sulfatide storage also occurs in visceral organs. In the gallbladder, metachromatic stroma macrophages often coexist with intestinal metaplasia of the epithelium, hyperplasia of the muscle wall and papillomatosis. Occurrence of gallbladder carcinoma has been reported in young MLD patients, suggesting that sulfatide accumulation at this site may predispose to development of neoplasia [258].

The disease mechanisms underlying MLD are only partly understood. ASA is necessary for catabolism of sulfatide to galactocerebroside via hydrolysis of the 3-O ester bond of galactosyl and lactosyl sulfatides [17]. Sulfatides are the most abundant sphingolipids in myelin, and have important functions in differentiation of myelinating cells, formation of paranodal junction, signaling at the plasma membrane, and myelin maintenance [51]. Astrocytes and neurons contain relatively low amounts of sulfatides. Their dramatic increase in MLD could lead to neuronal degeneration and astrocytic dysfunction. In vitro, sulfatide loading triggers the synthesis of inflammatory cytokines (TNF-α, IL-1β, IL-8) involved in recruitment of inflammatory cells and apoptosis [41, 121]. This suggests that sulfatide excess may induce and augment the inflammatory response contributing to oligodendrocyte and neuronal death. Ganglioside GD3 is highly expressed in activated microglia and reactive astrocytes, and could also play a role in apoptotic cell death of oligodendrocytes [199]. Finally, sulfatides trigger intracytoplasmic calcium accumulation with altered calcium homeostasis leading to cellular stress and apoptosis [43, 121].

At present, there is no general curative treatment available for all forms of MLD. The ideal therapy must provide persistent and high level expression of ASA in the CNS. Different therapeutic strategies have been developed and studied in animal models, and some have proceeded to clinical trials in MLD patients. Hematopoietic stem cell transplantation (HSCT) from bone marrow or umbilical cord blood provides monocytes that are able to cross the blood–brain barrier, differentiate into macrophages and deliver ASA to CNS resident cells to correct the enzyme deficiency. Replacement of resident tissue, however is slow, making HSCT ineffective for overtly symptomatic patients and for those with the most aggressive infantile-onset disease. In some, but not all juvenile and adult-onset patients, HSCT may delay or even halt disease progression and brain demyelination and sporadic patients have been reported [257] who showed improvement of clinical signs and white matter signal changes on MRI [253]. The cellular effects of HSCT on the neuropathology of MLD have still to be investigated. Enzyme replacement therapy administered intravenously is not effective because of inability of the enzyme to cross the blood–brain barrier. Gene therapy, based on genetic modification of autologous hematopoietic stem cells to express or over-express the ASA enzyme, is a promising option, but predictability of gene-transduction efficacy and engraftment of transduced cells must be optimized [18, 198].

### Astrocytopathies

Astrocytopathies are genetic white matter disorders due to mutations in astrocyte-specific gene products or in which astrocytes play a central role in the disease mechanisms. Increasing knowledge on the numerous roles of astrocytes in development, maintenance of homeostasis and response to injury suggest that disruption of normal astrocyte functions, astrocyte degeneration or dysfunctional maladaptive astrogliosis are the primary cause or the main factor in neurological dysfunction and disease [168].

#### Astrocytopathies due to mutations in astrocyte-specific gene products: Alexander disease

Alexander disease is a rare, untreatable and fatal genetic astrocytopathy. The age of onset varies from prenatal through adult forms. Patients are currently classified into two distinct disease categories depending on distribution of lesions and clinical presentation, with type I cases being early-onset and type II disease occurring at all ages [177]. Alexander disease is caused by dominant gain-of-function mutations in the astrocyte-specific cytoskeletal intermediate filament protein glial fibrillary acidic protein (*GFAP*) gene [25]. Most mutations occur de novo, but with better recognition of later-onset patients increasing numbers of families are being detected.

**Fig. 2:**
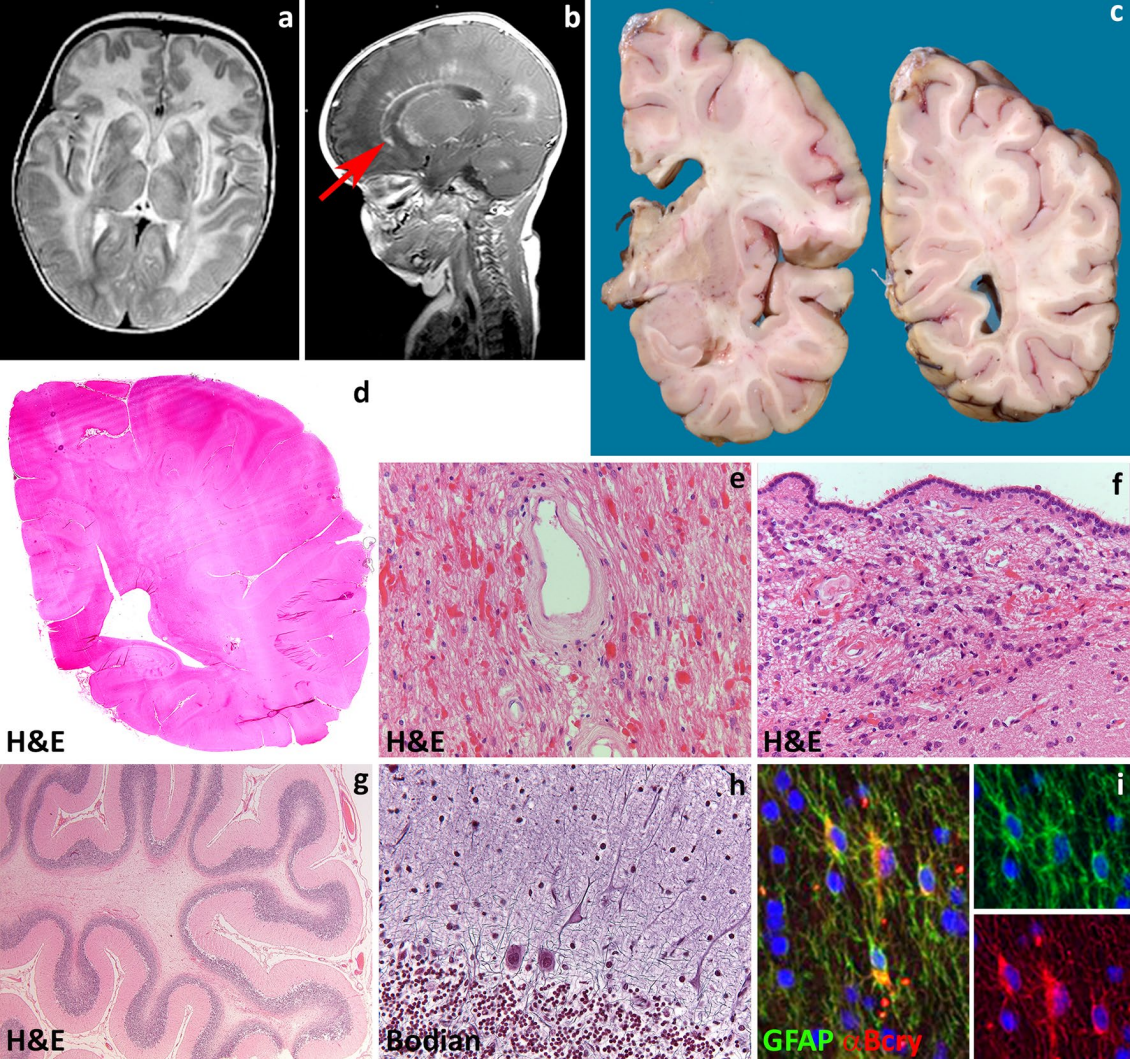
Alexander disease. **a** T2-weighted axial image of a 9-month-old infant shows a diffuse, bilateral and symmetric signal hyperintensity with a clear frontal predominance. The abnormal white matter is also moderately swollen. Around the ventricles is a rim of lower signal intensity. The basal nuclei and thalami are abnormal in signal. **b** T1-weighted axial image of the same child shows contrast enhancement along the wall of the lateral ventricle and head of the caudate nucleus (*arrow*). **c** Coronal sections through the cerebral hemisphere of a 9-year-old child show that the white matter is intact, but slightly *grayish*. **d** Haematoxylin & Eosin stain of a whole mount shows loss of staining distinction between gray and white matter. **e**, **f** Haematoxylin & Eosin stain shows abundance of Rosenthal fibers around white matter blood vessels (**e**) and along the wall of the lateral ventricle (**f**). **g** Haematoxylin & Eosin stain of a cerebellar folium shows mild cortical atrophy and intense white matter pallor. **h** Bodian stain of the cerebellar cortex shows swelling of the Purkinje cell dendrites. **i** Double fluorescence stain reveals that glial fibrillary acidic protein (GFAP)-positive astrocytes also strongly express the heat shock protein α-B crystallin (α-Bcry)

Most commonly, Alexander disease affects infants who present in the first years of life with developmental delay, spasticity, seizures and macrocephaly (type I disease). The disease is progressive, with most patients dying within 10 years from the onset. MRI shows extensive cerebral white matter changes (with high signal on T2- and low signal on T1-weighted images) with frontal predominance (Fig. 2a), a periventricular rim with high signal on T1-weighted images and low signal on T2-weighted images, signal abnormalities and possibly swelling of basal nuclei and thalami, brain stem abnormalities, and contrast enhancement of particular gray and white matter structures (Fig. 2b) [242]. Over time, tissue loss ensues with cystic degeneration of the frontal white matter, enlargement of the lateral ventricles, and cerebellar and brain stem atrophy. The basal nuclei and thalami also become atrophic with time. Later-onset patients (type II disease) commonly show insidious signs of hindbrain dysfunction such as ataxia, palatal myoclonus, dysphagia and dysphonia, frequently accompanied by spasticity and with eventual cognitive decline [139, 164, 246]. They have no macrocephaly. MRI shows brainstem predominance of lesions and atrophy, especially in the medulla oblongata and cervical spinal cord [56, 242, 246]. A kind of garlands can be seen along the lateral ventricles [246]. Contrast enhancement and cerebral white matter involvement may be absent [56].

Macroscopically, brains of infants with type I Alexander disease are enlarged and, on sectioning, show widespread lack of white matter in the cerebral hemispheres, cerebellum, brainstem and spinal cord (Fig. 2c, d). Degenerative changes are more prominent in the frontal white matter. Depending on survival, cortical thinning and atrophy of basal nuclei and thalami are also seen. Patients with type II disease have normal sized brains with atrophy of the caudal brainstem and cervical spinal cord and possibly patchy loss of myelin with gliovascular scarring in the cerebrum and cerebellum. Microscopically, the signature of Alexander disease is the widespread deposition of characteristic inclusions, known as Rosenthal fibers, in the setting of lack or loss of myelin (Fig. 2e, f). Rosenthal fibers are eosinophilic, refractile, rod- to oval-shaped inclusions located within the cell body and processes of perivascular, subpial, subependymal and white matter astrocytes. Rosenthal fibers are also present in the affected deep gray matter structures and brainstem and, to a lesser extent, in the neocortex where they accompany variable degrees of neuronal loss. Ultrastructurally, Rosenthal fibers appear as electrondense, osmiophilic structures surrounded by tangles of intermediate filaments [54]. In infants, the white matter contains little myelin and few, scattered oligodendrocytes. Phagocytes are not increased and no sudanophilic breakdown products are usually seen, compatible with a failure of myelin formation. In non-cystic areas, axons are preserved [24, 108, 150, 226, 267]. In childhood-onset cases, some degree of myelination occurs, as suggested by the relative preservation of U-fibers and presence of phagocytes containing neutral fats [24, 175, 190, 225]. Patients with later onset and brainstem and cerebellar signs can show Rosenthal fibers in the brainstem tegmentum and cerebellar cortex and deep white matter in addition to patches of myelin pallor and gliosis in the hemispheric white matter (Fig. 2g, h) [72].

Rosenthal fibers are composed of ubiquitinated aggregates of GFAP, the small heat shock proteins αB-crystallin (Fig. 4i) and Hsp27, vimentin, nestin, plectin, the 20S proteasome subunit, p-JNK, p62, and synemin [168]. Mutant GFAP aggregates in abnormal oligomers that cannot be assembled in the intermediate filament network and inhibit the proteasome [37, 39, 218, 219]. Mutant GFAP accumulation activates multiple stress pathways inside the astrocyte [218, 219]. Several lines of evidence suggest that these pathways are part of a stress response aiming at protecting the cell, that could theoretically be exploited for treatment purposes. A therapeutic role has been put forward for the transcription factor Nrf2 [120] and for αB-crystallin [168, 261]. αB-crystallin for example can disaggregate Rosenthal fibers in vitro [112] reducing the levels of toxic GFAP oligomers to produce monomers that can be degraded by the proteasome [217]. Additionally, constitutive overexpression of αB-crystallin in astrocytes results in a complete rescue from the otherwise lethal phenotype in a cross between two Alexander disease mouse models [77]. Which specific astrocytic functions are compromised in Alexander disease is still not known. Data suggest roles for abnormal expression of glutamate transporters [223] and for mislocalizaton and phosphorylation of the DNA- and RNA protein TDP43 [260]. In a mouse model of Alexander disease, adult hippocampal neurogenesis is severely compromised [78]; whether this also occurs in patients is unknown. Finally, the functional consequences of GFAP mutations on developmental myelination and myelin maintenance are obscure.

#### Astrocytopathies causing intramyelinic vacuolization: megalencephalic leukoencephalopathy with subcortical cysts

Megalencephalic leukoencephalopathy with subcortical cysts (MLC) is a myelin disorder characterized by chronic white matter edema with onset in infancy [202, 234]. MLC may present with two clinical phenotypes with different course. The classic progressive phenotype is most common and caused by recessive mutations in the *MLC1* (MLC1) [125] or the *GLIALCAM* genes (MLC2A) [130]. More recently, a different phenotype has been described characterized by early clinical features typical of MLC, but remarkable amelioration of symptoms over time (MLC2B) [239]. This remitting form of MLC is caused by dominant mutations in *GLIALCAM* [130, 236].

**Fig. 3:**
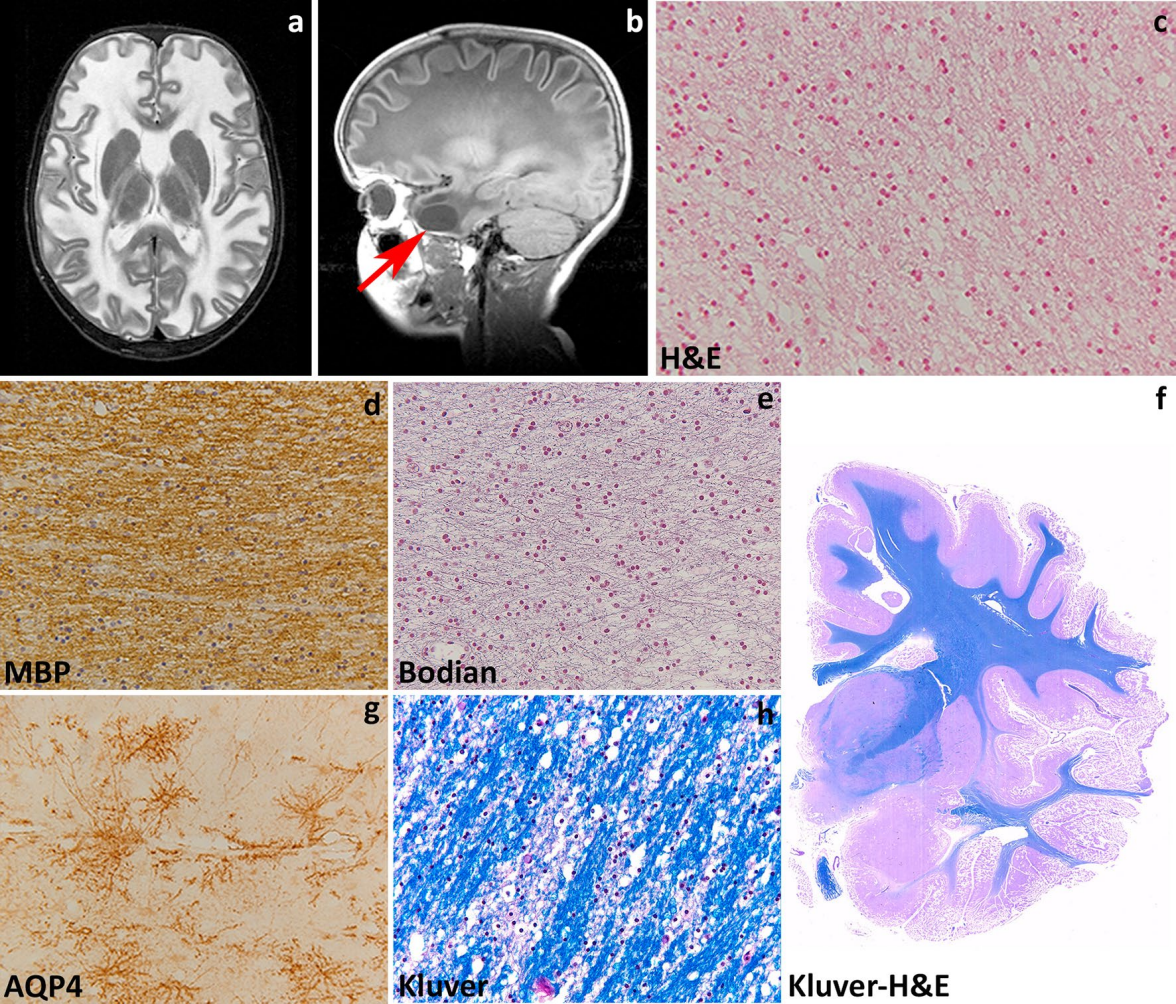
Megalecephalic leukoencephalopathy with subcortical cysts. **a** T2-weighted axial image of an 8-year-old child with *MLC1* mutations shows a diffuse, bilateral and symmetric signal hyperintensity in the cerebral white matter. The abnormal white matter is also mildly swollen. **b** T1-weighted sagittal image of the same child shows a subcortical cyst in the temporal pole (*arrow*). **c** Hematoxylin & Eosin stain of the subcortical white matter shows innumerable small vacuoles possibly crossed by thin tissue strands, indicative of intramyelinic oedema. **d** Stain against the major myelin protein myelin basic protein (MBP) shows normal amounts of myelin. **e** Bodian stain shows that axons are preserved. **f** Whole mount of a coronal section stained with Klüver-Haematoxylin & Eosin of a 30-year-old patient with a dominant *GLIALCAM* mutation shows complete integrity of the white matter. **g** In this patient, subcortical astrocytes strongly express the water channel Aquaporin 4 (AQP4), but the myelin amounts are normal and little intramyelinic oedema is present (**h**, Klüver stain)

Patients with MLC present with increasing macrocephaly in the first year of life. In the second year, head growth rate slows to normal and macrocephaly stabilizes [234]. After several years, slowly progressive cerebellar ataxia and spasticity develop with late mild cognitive decline. Most patients also have epilepsy. The clinical course is variably progressive: most children become wheelchair-bound as teenagers, but some patients remain paucisymptomatic as adults [236]. MRI reveals swelling and diffuse signal changes of the cerebral white matter from early infancy (Fig. 3a). The swelling is most severe in the first years of life and then slowly decreases [234, 236]. Diffusion parameters indicate that white matter water content is highly increased [251]. Subcortical cysts are invariably present in the anterior temporal region, frequently also in frontal and parietal regions (Fig. 3b). On follow-up, white matter atrophy ensues [234]. Patients with the remitting phenotype also develop macrocephaly within the first year of life. Their initial development is normal or mildly delayed. Subsequently, motor and cognitive capabilities often become normal and head circumference may normalize [239], although some patients have a cognitive deficit or autism. In these patients, striking improvement and normalization of the initial MRI abnormalities occur and subcortical cysts decrease in size and disappear.

Pathological data for MLC are extremely scarce with information being available only from one autopsy and four brain biopsies [23, 49, 86, 141, 165, 235]. Macroscopic features are not reported. Microscopic examination of the cortex only shows astrocytosis of the molecular layer, a finding relatable to chronic epilepsy [86, 235]. The white matter contains normal myelin content, but harbors countless small or larger vacuoles (Fig. 3c), the lining of which is immunopositive for myelin proteins [141, 235]. Electron microscopy confirms that the vacuoles are covered by membranes with a layered structure with major dense and intraperiod lines, confirming their intramyelinic location. The separation of the myelin lamellae occurs at the intraperiod line in the outer part of myelin sheaths. Vacuoles are also present in the astrocytic endfeet abutting on capillaries [49]. The white matter also shows fibrillary astrogliosis and little, if any, microglia cell activation [141, 235]. Extracellular spaces may be enlarged [86, 165], and myelin sheaths abnormally thin [86, 141, 165]. The amount of myelin is normal (Fig. 3e, f, h).

Insight in the disease mechanisms underlying MLC has greatly increased in the last decade also after development of MLC animal models recapitulating cardinal features of MLC. MLC1 is expressed in the CNS and to a much lesser degree in white blood cells [157]. In the CNS, MLC1 expression is only found in the cell membrane of white and gray matter astrocytes, especially those abutting the blood vessels and brain–cerebrospinal fluid barriers, and in ependymal cells and Bergmann glia in the cerebellum [23, 197, 221]. The astrocyte-exclusive expression of MLC1, together with some degree of ion channel homology of the protein [23, 125] and the highly increased white matter water content in MLC patients [236, 251], suggested that MLC1 is involved in regulation of ion–water homeostasis [125]. In agreement with this, depletion of MLC1 in cultured astrocytes causes disturbances in ion and water exchange during hypo-osmotic stress [50, 184]. Specifically, reduced MLC1 expression in astrocytes from MLC patients and *Mlc1*-null mice is associated with decreased volume-regulated anion channel (VRAC) chloride currents and reduced rate of the regulatory volume decrease after cell swelling. In line with a role for MLC1 in astrocytic volume regulation, *Mlc1*-null mice develop swelling of astrocyte perivascular endfeet and processes followed by water retention in the brain and spongiform myelin changes at later stages [50]. Moreover, *Mlc1*-null mice [50] and one patient brain [203] showed decreased expression of GlialCAM (encoded by *GLIALCAM*) and of the chloride channel ClC-2, also involved in ion–water homeostasis, and redistribution or increased expression of the potassium channel Kir4.1 [50] and the water channel aquaporin4 (Fig. 3g, unpublished). Functional interaction of MLC1 with other protein (β1 subunit of Na,K-ATPase, TRPV4, agrin and ZO-1) has been advocated in vitro [22, 26, 49, 100, 118, 130], but not confirmed in vivo [50]. Recent in vitro findings also suggest specific roles for MLC1 in astrocyte proliferation and maturation and in down-regulating astrocyte response to injury [119] via regulation of the epidermal growth factor receptor signaling.

The *MLC1* gene is present only in species that have myelin [23] and MLC1 expression is highest during active myelination in mice and men [50]. In both *Mlc1*-null mice and MLC patients, white matter edema is most pronounced when MLC1 expression levels are highest, indicating that white matter water retention correlates with lack of MLC1 function and explaining the decrease of edema at later stages [50].

GlialCAM is an immunoglobulin-like protein that functions as chaperone protein for MLC1. In the CNS, GlialCAM co-localizes with MLC1 [45, 130] in astrocytes, but is also found in axons and oligodendrocytes [57, 130]. *GLIALCAM m*utations affect trafficking of MLC1 to the cell membrane, explaining why recessive mutations in *MLC1* and *GLIALCAM* lead to MLC1 dysfunction and an indistinguishable clinical phenotype [130, 239]. Hence, a defect in MLC1 is the cardinal pathomechanism in the typical progressive form of MLC. The functional consequences of autosomal dominant *GLIALCAM* mutations are, however, still largely unclear [10]. The fact that GlialCAM is not obligatorily associated with MLC1 and that is expressed in cell types that do not express MLC1, as oligodendrocytes, suggest that it has other functions [131].

At present, there is no treatment for MLC. The existence of a remitting form of MLC, however, demonstrates that intramyelinic edema due to defects in ion–water homeostasis is potentially reversible.

#### Astrocytopathies determined by predominant astrocytic dysfunction: vanishing white matter

Vanishing white matter (VWM) is one of the more prevalent leukodystrophies [237]. It may present at any age from prenatal onset to senescence, with age at onset most often between 2 and 6 years [245], and is always fatal. VWM is also referred to as “Childhood ataxia with central nervous system hypomyelination” (CACH) [194] and “myelinopathia periaxialis diffusa” [28]. “Cree leukoencephalopathy” is a severe variant of VWM with onset in the first year of life and early death [21, 62]. VWM is caused by recessive mutations in any of the five genes (*EIF2B1*, *EIF2B2*, *EIF2B3*, *EIF2B4*, and *EIF2B5*) encoding the subunits of eukaryotic translation initiation factor 2B (eIF2B, subunits α, β, γ, δ and ε) [240]. eIF2B is a central regulator of translation initiation [176]. VWM shows a clear genotype–phenotype correlation [62, 249]. There is, however, wide phenotypic variability amongst patients with the same mutations, suggesting that other genetic and/or environmental factors influence the phenotype [249].

**Fig. 4:**
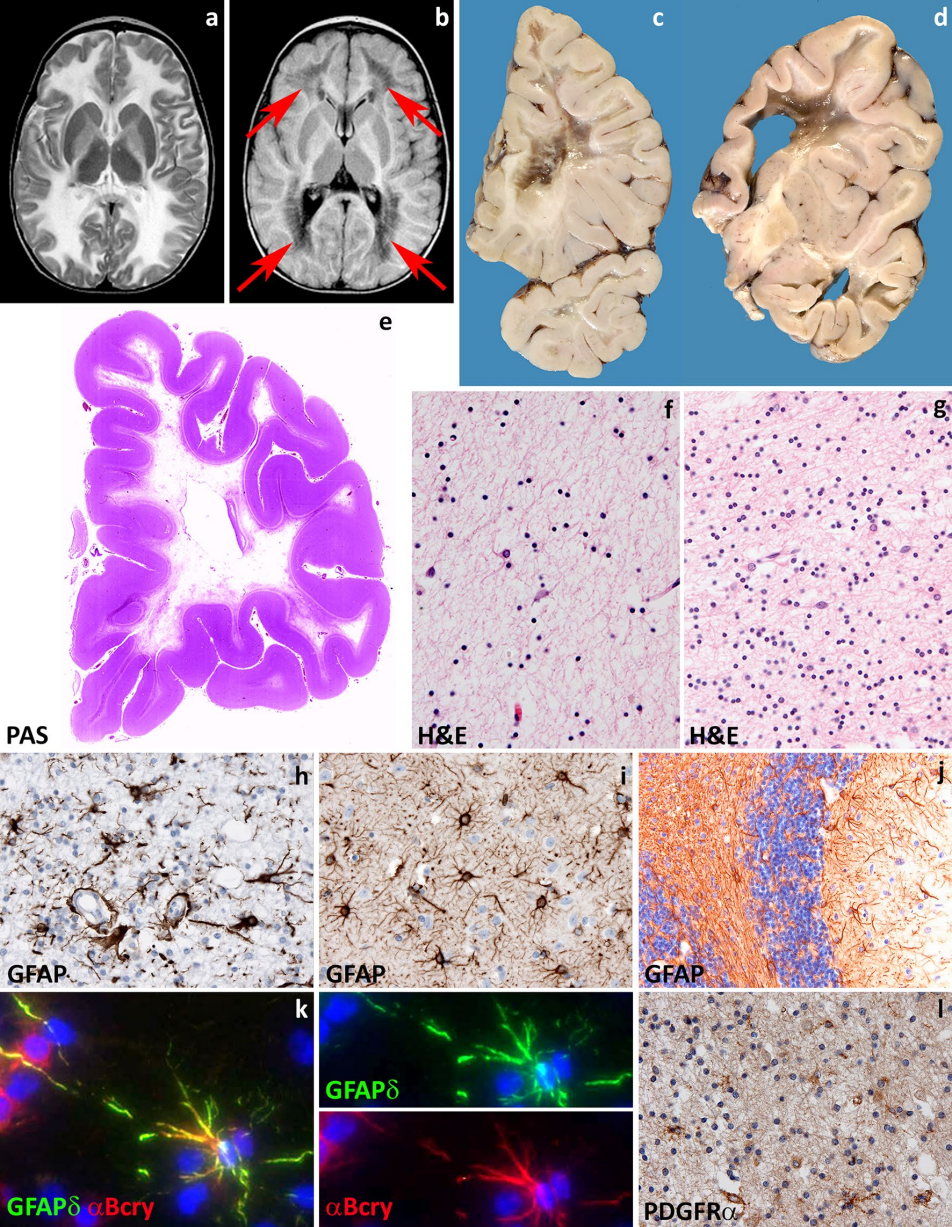
Vanishing white matter. **a** T2-weighted axial image of a 1-year-old child shows a diffuse, bilateral and symmetric signal hyperintensity in the cerebral white matter extending to the internal and external capsules. **b** Fluid attenuated inversion recovery axial image of the same child shows loss of tissue in the periventricular and deep white matter (*arrows*). **c**, **d** Coronal cut of the brain of a 10- and a 6-year-old child confirms loss of the deeper cerebral white matter to a variable degree. The residual white matter appears grayish and gelatinous. There is a relative sparing of the internal capsule (**d**) and, in places, of the U-fibers. **e** Whole mount of a coronal section of a 10-year-old child stained with Haematoxylin & Eosin shows diffuse white matter rarefaction and cystic degeneration. **f** Haematoxylin & Eosin stain of the more affected frontal white matter shows marked tissue rarefaction with scarcity of astrocytes. **g** Haematoxylin & Eosin stain of the relatively spared cerebellar white matter shows some degree of tissue vacuolization and increased cellularity. **h** Stain against the glial fibrillary acidic protein (GFAP) of the more severely affected white matter shows dysmorphic astrocytes with short, blunt cell processes. **i** In the unaffected cerebral cortex, GFAP stain shows astrocytes with normal morphology. **j** In the cerebellar cortex, GFAP stain shows mislocalization of Bergmann glia to the molecular layer. **k** Double fluorescent stain shows that astrocytes robustly express the delta isoform of GFAP (GFAPδ) and the heat shock protein α-B crystallin (α-Bcry). **l** Stain against the oligodendrocyte precursor marker PDGFRα shows abundance of immunopositive cells in the relatively spared white matter

Age at onset in VWM predicts disease severity and survival [245]. Young children with classical VWM develop progressive neurological deterioration with cerebellar ataxia, less prominent spasticity and relatively mild cognitive deterioration [84, 194, 233, 238, 245]. Optic atrophy and epilepsy may also occur. Adult patients often present with presenile [62] dementia, psychiatric symptoms or complicated migraine. The disease progresses slowly with superimposed episodes of rapid major deterioration following stresses as febrile infections and minor head trauma [233, 238, 245]. These episodes may end in coma and death. Prenatal forms of VWM show primary microcephaly and signs of extraneurologic involvement in addition to ovarian dysgenesis and leukoencephalopathy [247]. After infancy, primary or secondary premature ovarian failure is a common concurrent sign that may even precede the neurologic decline, a condition referred to as “ovarioleukodystrophy” [61, 195, 233]. MRI is typically characterized by progressive rarefaction with cystic degeneration of the cerebral white matter. Diffuse white matter signal abnormalities are already present before the onset of symptoms [233, 248]. With time, the affected white matter disappears and is replaced by fluid (Fig. 4a, b) [233, 238, 245]. Radial stripes extending from the ventricular wall to the subcortical regions are often visible, suggesting remaining tissue strands [233, 238, 245]. There is no contrast enhancement. The cerebellar white matter is often mildly abnormal, but not cystic, and the central tegmental tracts at the level of the pons are characteristically involved [233, 238]. The cerebral cortex is always spared, whereas the thalamus, midbrain and pons may be involved [233, 238, 245]. The spinal cord is usually spared [238, 245].

Macroscopically, the brains of children with classical VWM are generally of normal size. Some degree of brain swelling is common in neonates and infants, while cortical and subcortical atrophy are common in adults [29]. On sectioning, the cerebral white matter is grayish and appears gelatinous, cystic or frankly cavitated especially in the frontoparietal deep regions (Fig. 4c, d, e). Cerebellar and brainstem white matter is much less involved, whereas the optic system, anterior commissure, corpus callosum and internal capsules are characteristically spared. U-fibers are also relatively spared [29]. Gray matter structures are usually unaffected; however, basal ganglia and cerebellar cortex can be atrophic and the ventricles enlarged [209]. The spinal cord is most often spared [4, 71, 233]. Microscopically, the affected white matter shows lack of myelin, myelin vacuolation, cystic changes, and only rarely loss of myelin with macrophages, arguing against demyelination [29]. The most striking histopathological changes are typically seen in oligodendrocytes and astrocytes. Oligodendrocyte numbers are reduced in the cavitated lesions [28, 270], but markedly increased in the U-fibers and in relatively spared white matter areas (Fig. 4f,g) [30, 65, 73, 186, 254, 263]. “Foamy” vacuolated oligodendrocytes have also been described [62, 270] and considered by some to be a specific marker for VWM; however, they are not consistently detected [62]. Electron microscopy reveals that vacuoles in foamy oligodendrocytes are membranous structures associated with mitochondrial membranes and contiguous with myelin lamellae [270]. Both foamy and normal oligodendrocytes contain many mitochondria and fingerprint structures [73, 238, 270]. Myelin sheaths are thin or absent and in relatively spared white matter areas vacuolated myelin is noted reflecting intramyelinic edema [186, 194, 233, 238, 270]. Reactive astrogliosis and microglia cell activation are characteristically meager in VWM, even in areas near the cavitation. Astrocytes are reduced in number and dysmorphic with broad blunt processes instead of their typical delicate arborizations (Fig. 4h). Dysmorphic astrocytes are present in the cerebral while matter, but only sparse in the relatively spared cerebellum and typically absent in gray matter structures (Fig. 4i) [32]. As an exception, cerebellar cortical Bergmann glia are typically mislocalized to the molecular layer (Fig. 4j) [57]. There is variable loss of axons often with axonal thinning and axonal swelling and spheroids [63, 174, 186, 194, 238, 270]. The gray matter is spared or greatly preserved, but mild astrocytosis and microgliosis may be detected. The neuropathologic features of Cree leukoencephalopathy are similar, however, without oligodendroglial hypercellularity and astroglial abnormal morphology [21, 62]. Systemic findings are nonspecific [247].

**Fig. 5:**
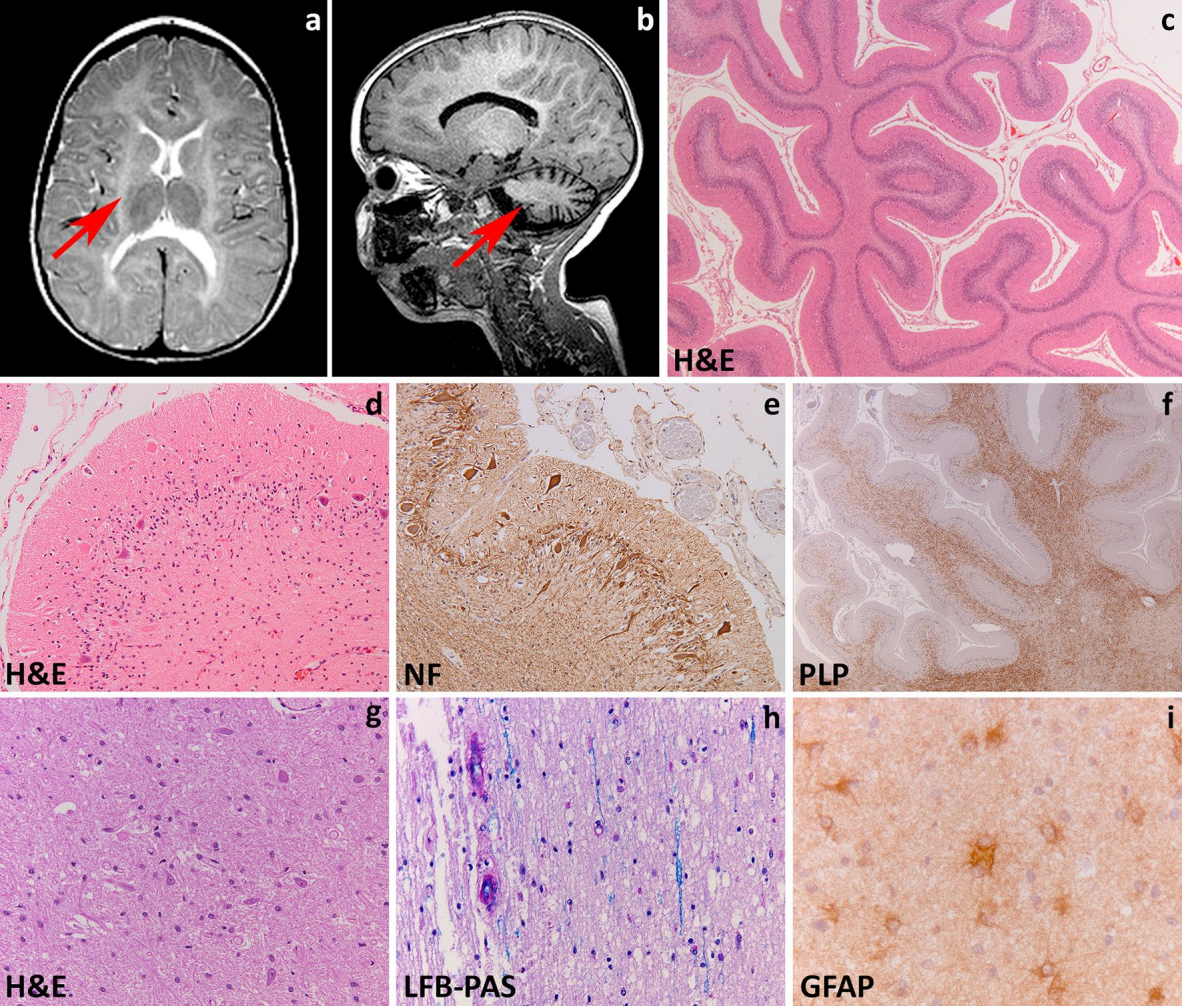
Hypomyelination with atrophy of the basal ganglia and cerebellum. **a** T2-weighted axial image of a 3-year-old child shows a mild, diffuse, bilateral and symmetric signal hyperintensity in the cerebral white matter. Note that the putamen has virtually disappeared (*arrow*). **b** T1-weighted sagittal image of the same child shows moderate cerebellar atrophy (*arrow*). The white matter is T1 hyperintense. The combination of mild T2 hyperintensity and T1 hyeprintensity is compatible with mild hypomyelination. **c** Haematoxylin & Eosin stain of a cerebellar folium shows cortical atrophy with thinning of the cortical granular layer. **d** Haematoxylin & Eosin stain of the cerebellar cortex reveals that loss of granular neurons may be severe and also accompanied by drop out of Purkinje cells. **e** Stain against neurofilaments (NF) shows swelling of cerebellar cortical Purkinje cells axons and dendrites. **f** Stain against the major myelin protein proteolipid protein (PLP) shows patchy lack of myelin in the cerebellar white matter. **g** Haematoxylin & Eosin stain of the caudate nucleus shows mild loss of neurons; the putamen could not be identified. **h** Luxol fast blue-Periodic acid Schiff (LFB-PAS) stain of the deeper white matter shows severe lack of myelin with decreased cellularity reflecting oligodendrocyte loss. **i** Stain against the glial fibrillary acidic protein (GFAP) of the white matter shows mild reactive gliosis with dividing astrocytes

Several lines of evidence support the notion that astrocytes are a central determinant in the pathogenesis of VWM. In cell cultures of human glial progenitors with a defect in *EIF2B5*, astrocyte generation was compromised and cells had an abnormal morphology [47]. In vivo, dysmorphic astrocytes are immature cells with the immunohistochemical profile of astrocyte precursor cells [32, 48]. Dysmorphic astrocytes characteristically overexpress the delta isoform of GFAP (GFAPδ), but not the major isoform GFAPα or total GFAP (Fig. 5k) [30]. This indicates that the intermediate filament network is compromised in VWM astrocytes, and may explain their abnormal morphology and the lack of reactive gliosis. In recently developed mouse models of VWM, Bergmann glia in the cerebellum and Muller cells in the retina are also affected. In particular, Müller cell abnormalities are associated with retinal dysplasia. Retrospective evaluation of patients’ electroretinographic data confirmed that retinopathy is also a sign of human VWM [48]. VWM astrocytes also impact on oligodendrocyte maturation. In patients’ brains, a large portion of white matter oligodendrocytes are OPCs that proliferate, but fail to mature into myelin-forming cells (Fig. 5l) [30] and possibly die by apoptosis [28, 254]. Co-cultures of VWM and wild-type mouse OPCs and astrocytes in different combinations proved that VWM astrocytes impede wild-type OPC maturation whereas mutant OPCs mature normally when cultured with wild-type astrocytes [48]. Overall, these data suggest that VWM is a developmental disorder of glia cells driven by astrocytic pathology.

How eIF2B mutations determine astrocytic dysfunctions is still unknown. eIF2B plays a key role in translation initiation, in which ribosomes are assembled on mRNA [107]. This occurs via delivery by eIF2 of the initiator methionyl-transfer RNA (Met-tRNAi) to the small ribosomal subunit. When the start codon is recognized, the eIF2-bound guanosine triphosphate (GTP) is hydrolyzed to inactive guanosine diphosphate (GDP)-bound eIF2. In order to bind another Met-tRNAi, active eIF2 must be regenerated by exchange of GDP for GTP. This step is catalyzed by the guanine nucleotide-exchange factor eIF2B and necessary for each round of translation initiation [176]. eIF2B activity, thus, regulates global rates of protein synthesis [89]. Upon stress, protein synthesis is inhibited. Stress may lead to misfolding and denaturation of proteins, contributing to cell dysfunction and death. Various stimuli, including thermal, chemical, oxidative or physical trauma, inhibit protein synthesis within a cell protective mechanism called the cellular stress response [264]. In VWM brains, this response is constitutively activated [256]. In VWM patients, serious deteriorations often follow head trauma and febrile infections, an observation that could correlate with the regulating role of eIF2B on translation upon stress. The functional effects of VWM mutations on eIF2B activity are, however, very diverse, and some mutations do not have any effect, even though they cause severe disease [128]. Decreased eIF2B activity does not impact global protein synthesis, regulation of protein synthesis upon stress or viability of patients’ cells [101, 113, 256]. It is still unclear why, amongst the organs with high metabolic rate, the brain is selectively affected.

At present, there is no effective treatment for VWM, except prevention of known stress conditions that may provoke an episode of deterioration.

### Leuko-axonopathies

Leuko-axonopathies are genetic white matter disorders due to defects in neuron- or axon-specific gene products or in which the central disease mechanisms can be conducted back to axons. Distinguishing microscopically primary axonopathies with secondary myelin pathology from myelin disorders with secondary axonal loss is challenging, a fact that may have contributed to the long-lasting under-recognition of leuko-axonopathies. MRI pattern recognition combined with next generation sequencing [196], however, has dramatically increased the number of white matter disorders linked to gene products expressed also or solely in neurons and axons. Interestingly, many are characterized by hypomyelination and include amongst others disorders due to mutations in *AGC1* (global cerebral hypomyelination) [265, 268], *HSPD1* [134], *HCC* (hypomyelination and congenital cataract) [66], and *AIMP1* [58].

#### Leuko-axonopathies due to mutations in axonal gene products: hypomyelination with atrophy of the basal ganglia and cerebellum

Hypomyelination with atrophy of the basal ganglia and cerebellum (H-ABC) is a rare childhood neurodegenerative disease characterized by extrapyramidal movement disorders, spasticity, and cerebellar ataxia [244]. H-ABC is due to dominant, most often de novo mutations in the *TUBB4A* gene [80, 200]. *TUBB4A* encodes tubulin β-4A, a principal constituent of microtubules highly expressed in the brain [80]. *TUBB4A* mutations are also associated with other neurological disorders, including dystonia type 4 (DYT4), and isolated hypomyelination. DYT4 presents with adolescent- or adult-onset generalized dystonia, whispering dysphonia and normal brain MRI [88, 129, 266]; isolated hypomyelination is characterized by slowly progressive ataxia and spasticity, and deficient myelination with variable cerebellar atrophy on MRI [172]. This indicates a disease continuum associated with changes in *TUBB4A*, most likely reflecting a genotype–phenotype correlation [80].

Patients with classical H-ABC present in the first few years of life with developmental delay, hypotonia, nystagmus and deterioration of motor functions with spasticity and cerebellar ataxia. Extrapyramidal movement disorders, including dystonia, rigidity and possibly choreoathetosis and perioral dyskinesias are an almost invariably accompanying sign. Epilepsy with microcephaly and stunted growth may also be present. Signs of bulbar dysfunction are a common complaint, including dysphonia, dysarthria and difficulties swallowing. Cognition and language are variably impaired. The disease runs a slowly progressive course and is ultimately fatal [80, 146, 244]. MRI typically shows the triad of hypomyelination, atrophy of the basal nuclei (specifically the neostriatum) and cerebellar atrophy (Fig. 5a, b) [80, 244]. Hypomyelination, also evident in the corpus callosum, brainstem and cerebellum, may be severe and is followed in time by signs of myelin loss and white matter atrophy [241]. The neostriatum is often already atrophic in the first imaging studies and tends to become completely atrophic in the course of the disease. The globus pallidus and thalamus are typically unaffected. Cerebellar atrophy is often also already visible at onset, and can involve the vermis more than the cerebellar hemispheres.

Macroscopic examination shows atrophy of the cerebellum and to a lesser degree the brainstem. On sectioning, caudate and putamen are thinned and lateral ventricles are enlarged. The cerebral white matter appears slightly grayish, but has a normal consistence. Microscopically, the cerebellar cortex shows atrophy of the granular and molecular layer (Fig. 5c). There is some loss of Purkinje cells, with the remaining Purkinje cells showing swollen dendrites and axons (Fig. 5d, e) [241] [personal observation]. The white matter contains little myelin (Fig. 5f). The putamen is usually virtually disappeared with few, if any remaining neurons and robust astrogliosis. The caudate also shows slight neuronal loss and mild astrogliosis (Fig. 5g). The thalamus and globus pallidus are intact. Microscopy of the cerebrum shows marked lack of myelin in the deeper and subcortical cerebral white matter, extending to the U-fibers, with severe loss of oligodendrocytes (Fig. 5h). Some macrophages can accrue around blood vessels, indicating that the lack of myelin is related to both hypomyelination and myelin degeneration. Microglia cell activation and isomorphic reactive astrogliosis are accompanying features (Fig. 5i). Axons are better preserved, but axonal spheroids are always present. The cerebral cortex is normal.

Microtubules serve essential cellular activities that are critical for brain development and function. They provide structure and generate forces needed by neurons to migrate and develop axonal and dendritic processes, and provide organized scaffolds for motor proteins. An essential feature is their dynamic instability that is the possibility to rapidly de- and repolymerize in response to the environment [143]. Microtubules are assembled copolymers formed by alternating α- and β-tubulin subunits. Mutations in genes encoding the α- and β-tubulin isotypes may alter the dynamic properties and functions of microtubules in different ways and are linked to complex developmental disorders, including malformations of cortical development, schizencephaly, abnormalities of midline commissural structures and dysmorphisms of basal ganglia and hind-brain [188]. Albeit these “tubulinopathies” are malformative in nature, H-ABC is a degenerative disease and its pathogenesis is still a matter of speculation. H-ABC causing mutations could affect heterodimerization or polymerization altering microtubule dynamics or stability. In turn, this could hamper axonal transport leading to axonal dysfunction and loss [80, 155]. In line with this hypothesis, axonal spheroids are easily detected in the white matter of H-ABC patients. Early-onset neuronal and axonal dysfunction impairs developmental myelination and is typically associated with oligodendrocyte loss in the white matter [33, 75, 250], as observed in H-ABC. Ongoing axonal dysfunction is associated with myelin loss. Additionally, transport of myelin proteins as PLP and myelin basic protein to the myelin sheaths is microtubule-dependent [19, 35, 227], a mechanism which could also contribute to deficient myelin deposition and maintenance. Disrupted axonal transport may theoretically also account for the neuronal dropout in basal nuclei and cerebellar cortex [80].

No curative treatment for H-ABC is at present known.

#### Leuko-axonopathies in the context of early-onset neuronal degeneration: GM1 gangliosidosis

GM1 gangliosidosis is an autosomal recessive lysosomal storage disorder characterized by variable degrees of neurodegeneration and visceral and skeletal abnormalities. It is due to mutations in the *GLB1* gene resulting in decreased activity of the lysosomal enzyme acid β-galactosidase. This leads to accumulation of GM1 ganglioside substrates in lysosomes in the CNS, bones and visceral organs. Clinically, GM1 gangliosidosis shows a continuum of clinical presentations from a severe infantile form to a milder, chronic adult form, with an inverse correlation between disease severity and residual enzymatic activity [215].

**Fig. 6:**
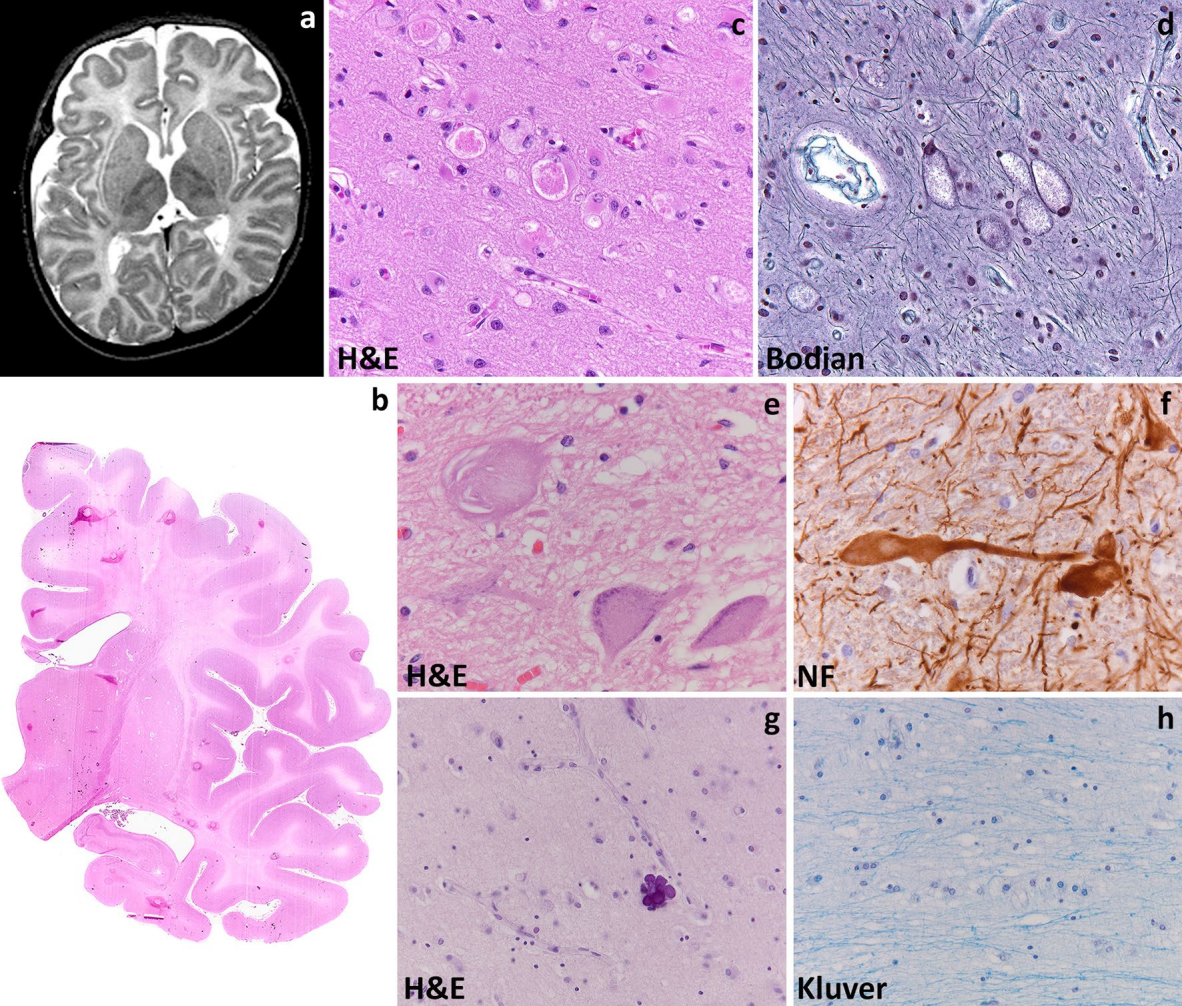
GM1 gangliosidosis. **a** T2-weighted axial image of an 8-month-old infant shows a diffuse, bilateral and symmetric signal hyperintensity in the cerebral white matter extended to the U-fibers, but sparing the internal capsule. Note the mild signal abnormality of the basal nuclei and thalami. **b** Whole mount of a cerebral coronal section stained with Haematoxylin & Eosin shows diffuse cortical atrophy, and white matter pallor and atrophy with enlarged lateral ventricle and thinning of the corpus callosum. **c** Haematoxylin & Eosin stain of the frontal cortex shows accumulation of storage material in the cytoplasm of some neurons and reactive gliosis. **d** Bodian stain of the thalamus shows neuronal storage with ballooned cells also at this site. **e** Haematoxylin & Eosin stain of the anterior horn in the cervical spinal cord shows neuronal storage in the alpha motor neurons. **f** Stain against neurofilaments (NF) shows that the neuronal storage may be prominent at the level of the axon hillock, giving rise to meganeurites. **g** Haematoxylin & Eosin stain of the deep cerebral white matter shows lack of myelin and paucity of oligodendrocytes. **h** In the same area, a Klüver stain confirms the lack of myelin

The type I, infantile form of GM1 gangliosidosis has onset in the first six months of life. It is characterized by rapid psychomotor deterioration and severe CNS involvement with spasticity, deafness, blindness, and decerebrate rigidity. Hepatosplenomegaly, coarse facial features, macular cherry-red spots, and skeletal dysplasia are also present, and death supervenes within ages one and three years. MRI shows diffuse white matter signal changes that are too marked to be only ascribed to delayed myelination (Fig. 6a). The cerebellar white matter is also involved, whereas the brainstem and corpus callosum appear better myelinated. Subtle signal changes are also visible in the thalamus and basal nuclei. Type II GM1 gangliosidosis, or late-infantile/juvenile form, has onset between six months and two years. It presents with psychomotor deterioration, progressive dementia, spasticity and cerebellar ataxia, extrapyramidal signs and epilepsy. Skeletal dysplasia may be associated, but hepatosplenomegaly and cherry-red spots are usually absent. MRI shows progressive global atrophy accompanied by subtle white matter signal abnormalities. Patients may survive into childhood. Type III GM1 gangliosidosis, the adult/chronic form begins in the second to third decade of life and is characterized by localized skeletal involvement and cardiomyopathy. CNS involvement is focal, usually presenting as dystonia or gait or speech disturbance. Epilepsy may occur, and cognition is impaired. MRI reveals signal changes and atrophy of the caudate nucleus and putamen, mild diffuse cerebral atrophy and subtle signal changes in the white matter [167, 182, 183, 232].

Macroscopically, patients with type I and II GM1 gangliosidosis may have a diffusely atrophic brain. On sectioning, the white matter has an increased consistency to the touch and is atrophic with enlarged lateral ventricles (Fig. 6b) [116, 212, 213, 215]. Adult type III patients often only show atrophy of the basal nuclei [72, 272]. Microscopic examination of type I GM1 gangliosidosis brains reveals a typical neuronal storage disease, with virtually all neurons showing enlarged ballooned cell bodies filled with vacuolated, PAS-positive material (Fig. 6c–e). Ganglioside storage is prominent at the axonal hillock region giving rise to meganeurites (Fig. 6f). Storage material is also detected in glia cells. At the ultrastructural level, inclusions appear as packed concentrically arranged lamellar structures enclosed within a lysosomal membrane, the so-called ‘membranous cytoplasmic bodies’. In long surviving cases, there is extensive neuronal loss. Early myelinated structures have usually normal myelin amounts, whereas areas last to be myelinated display profound lack of myelin and decreased oligodendrocyte numbers as a consequence of apoptotic cell death (Fig. 6g, h) [250]. This is accompanied by profound reactive gliosis, with or without axonal degeneration. Overall the white matter pathology is suggestive of a combination of deficient myelin deposition and myelin degeneration. In types II and III GM1 gangliosidosis there is an identical, although less diffuse neuronal storage. In type III in particular, the storage is most pronounced in the head of the caudate nucleus and anterior putamen. The dendrites of the cerebellar Purkinje cells are also filled with storage material, but this is virtually absent in the cerebral cortex. White matter involvement is either absent or minimal. The degree of visceral storage varies considerably, being diffuse and massive in infantile GM1 gangliosidosis and limited to absent in later-onset forms.

Gangliosides are normal components of cell membranes, particularly neurons in the regions of nerve endings and dendrites, and GM1 is the major ganglioside in the vertebrate brain. Due to reduced normal degradation, GM1 ganglioside and its asialo-derivative accumulate in lysosomes. Accumulation of toxic asialo-compound and lyso-compound GM1 ganglioside derivatives is believed to be neuropathic [95, 154, 215], resulting in neuronal dysfunction and eventually death. Additional factors contribute to the disease pathogenesis, including misregulation of proteins and intracellular trafficking, depletion of precursor pools, altered membrane composition and function, mislocalized storage compounds that activate the unfolded protein response (UPR) and trigger neuronal apoptosis [222], and altered regulation of endolysosomal proteins and enzymes by increased lipid load [192]. In addition, formation of meganeurites and increase in synaptic spines may disturb neuronal connectivity [178].

At present, there is no curative treatment for GM1 gangliosidosis. Enzyme replacement, substrate reduction, and anti-inflammatory treatments alone or in combination have shown improvement in symptoms and lifespan in some animal models, but not in patients. More recently, gene therapy has also shown results in animal models. Treatment with glucose analogues reducing the amount of ganglioside substrate is used as off-label treatment for some patients. Therapies under clinical development also include small molecule chaperones and gene therapy [182].

### Microgliopathies

Microgliopathies are white matter disorders due to defects in microglia-specific gene products or in which microglial dysfunction is at the center of the disease process [193]. In the healthy white matter, microglia exert roles in development, maintenance of homeostasis and function. Neuropathology has shown that almost no neurological diseases exist without microglial activation, which may play a central role in disease initiation and progression. In many conditions activated microglia secrete inflammatory cytokines, but microglial activation is not always associated with neuroinflammation. Conditions in which microglia activation is negligible could be envisioned as cases of pathological asthenia of this cell type. Microglia are also involved in repair. The almost universal reaction of microglia upon CNS injury and their roles in repair suggests that future knowledge will elongate the list of microgliopathies, including white matter disorders.

#### Microgliopathies due to mutations in microglia-specific gene products: hereditary diffuse leukoencephalopathy with axonal spheroids

Hereditary diffuse leukoencephalopathy with axonal spheroids (HDLS) is a rare, adult-onset neurodegerative disease [5, 207]. HDLS is due to autosomal dominant mutations in the *CSF1R* gene encoding the colony stimulating factor 1 receptor [179]. CSF1R is specifically expressed by microglia. More recently, *CSF1R* mutations were also identified in a clinically distinct adult-onset white matter disorder, pigmented ortochromatic leukodystrophy (POLD), suggesting that HDLS and POLD are the same disease entity [153].

**Fig. 7:**
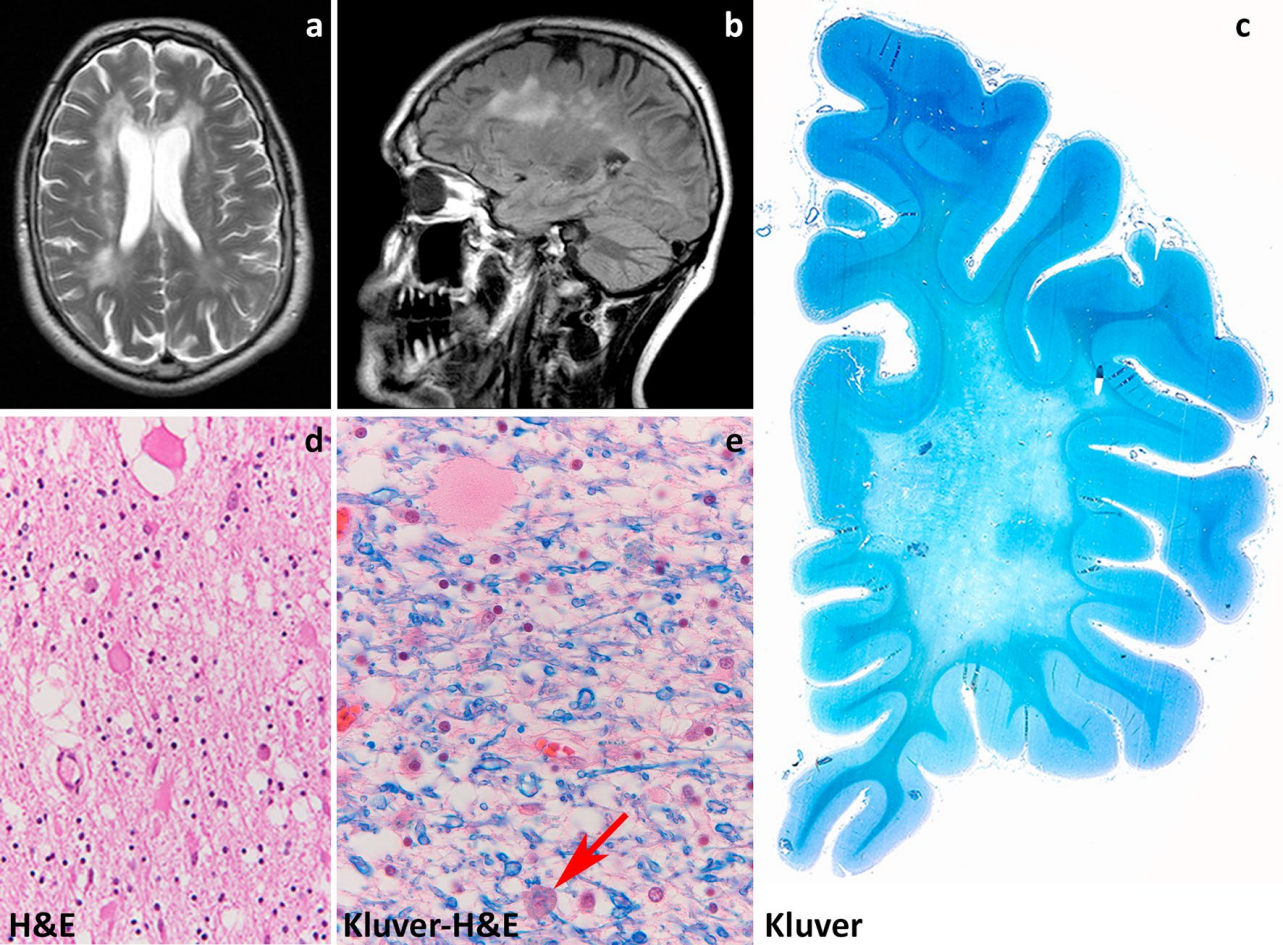
Hereditary diffuse leukoencephalopathy with axonal spheroids. **a** T2-weighted axial image of a 50-year-old patient shows patchy, bilateral signal hyperintensities in the periventricular and deep cerebral white matter with posterior–frontal preponderance. **b** Patchy signal changes are also visible in a fluid attenuated inversion recovery sagittal image of the same patient. Note also the slight cerebral atrophy with widening of the sulci. **c** Whole mount of a cerebral coronal section stained with a Klüver shows confluent lack of myelin of the deep white matter with relative sparing of the U-fibers. **d** Haematoxylin & Eosin stain of the frontal white matter shows tissue rarefaction and axonal spheroids. **e** Klüver-Haematoxylin & Eosin stain of the parietal white matter shows lack of myelin, an axonal spheroid and a pigmented cell (*arrow*)

Most often, patients with HDLS present in their third to fifth decade of life with progressive personality changes, dementia, spasticity, gait difficulties and depression [207, 210]. Parkinsonism and epilepsy may also occur [210]. The clinical course is rapidly progressive and the disease is invariably fatal. The phenotype is, however, heterogeneous with significant intra- and inter-familial variability of clinical features and disease duration [207]. MRI shows bilateral, patchy T2-hyperintense signal changes in the periventricular, deep and subcortical cerebral white matter that are usually asymmetric and spare the U-fibers (Fig. 7a, b) [243]. The frontal and parietal regions are earlier and more severely affected. White matter atrophy with enlargement of the lateral ventricles and thinning of the corpus callosum also are features. The cerebellar white matter is minimally affected. There may be atrophy of the cerebral cortex, but the deep gray matter structures and brainstem are usually spared [211, 243]. In some patients, the affected cerebral white matter also contains spotty calcifications [111]. MRI changes are progressive [211].

Macroscopically, the brains of HDLS patients may show slight cortical atrophy in the anterior regions. On section, the white matter is atrophic with possibly some degree of softening and grayish discoloration (Fig. 7c). Microscopically, the affected white matter is rarefied and vacuolated with loss of myelinated fibers. The hallmark of the disease is the diffuse presence within the white matter lesions of swollen axons and axonal spheroids that are typically immunopositive for the phosphorylated neurofilaments and amyloid precursor protein stains (Fig. 7d, e) [5, 6, 83, 210, 243]. Accumulation of lipid-laden macrophages, microglia cell activation and reactive astrogliosis with bizarre morphologies are also seen [5, 6, 210, 243]. Microglia in HDLS display key features that can be exploited for the neuropathological diagnosis. Activated microglia are typically spatially restricted rather than diffusely distributed, show a characteristic morphology with thin processes and many knot-like structures, and are smaller than those in other CNS diseases including X-linked adrenoleukodystrophy [111, 160, 185]. Additionally, pigmented glia are present, a feature also characterizing POLD (Fig. 7e) [136]. The neocortex is usually unremarkable. In the lower layers of the cortical areas overlying the white matter pathology, however, there may be ballooned neurons [6]. Mild neuronal loss, reactive gliosis and sparse spheroids can also be noted in the basal nuclei. The long tracts in the brainstem, including the cortico-spinal tracts, may be affected [210, 243].

CSF1R is the receptor for colony stimulating factor 1, a cytokine controlling production, differentiation and chemotaxis of mononuclear phagocytic cells and supporting their activation [171]. In the CNS, CSF1 and its receptor, CSF1R, play a key role in maintaining microglial homeostasis [171]. CSF1R virtually mediates all biological effects of the cytokine. The encoded protein is a tyrosine kinase transmembrane receptor, and all HDLS-causing mutations are located in the tyrosine kinase domain of the protein [179]. Ligand binding activates the receptor kinase through a process of oligomerization and transphosphorylation. In vitro functional expression studies of some HDLS-causing mutations showed that the mutant proteins are not able to autophosphorylate, suggesting a defect in kinase activity that would abate downstream signaling [111, 153, 179].

The mechanisms by which CSFR1 dysfunction leads to impairment of white matter maintenance are still largely obscure. Some suggestion, however, exists that myelin loss may be precociously accompanied or even preceded by axonal pathology. Three lesion stages may be recognized in HDLS: the presence of axonal spheroids within well-myelinated white matter, the presence of axonal spheroids on a background of loss of myelin, and confluent axonal and myelin loss [3].

### Leuko-vasculopathies

Leuko-vasculopathies or leuko-micro-angiopathies are genetic white matter disorders in which the main disease mechanisms involve the brain small blood vessels. The term small vessel disease refers to pathological processes with different etiologies affecting small arteries, arterioles, venules and capillaries [162]. The consequences on the brain parenchyma are mainly lesions located in the subcortical regions, including white matter lesions, lacunar infarcts, and hemorrhages. As a whole, small vessel diseases are a leading cause of functional loss and cognitive decline [162]. Genetic forms are rare, often transmitted as an autosomal dominant trait and typically have onset in adulthood. Amongst other forms, they include autosomal dominant arteriopathy with subcortical infarcts and leukoencephalopathy (CADASIL), cerebral autosomal recessive arteriopathy with subcortical infarcts and leukoencephalopathy (CARASIL), and hereditary cerebral amyloid angiopathy [162]. Especially in the older adult and elderly populations the common association with systemic and life-style risk factors as hypertension, diabetes and smoking suggests that genetic leuko-vasculopathies may be underdiagnosed [187].

#### Cathepsin A-related arteriopathy with strokes and leukoencephalopathy

**Fig. 8:**
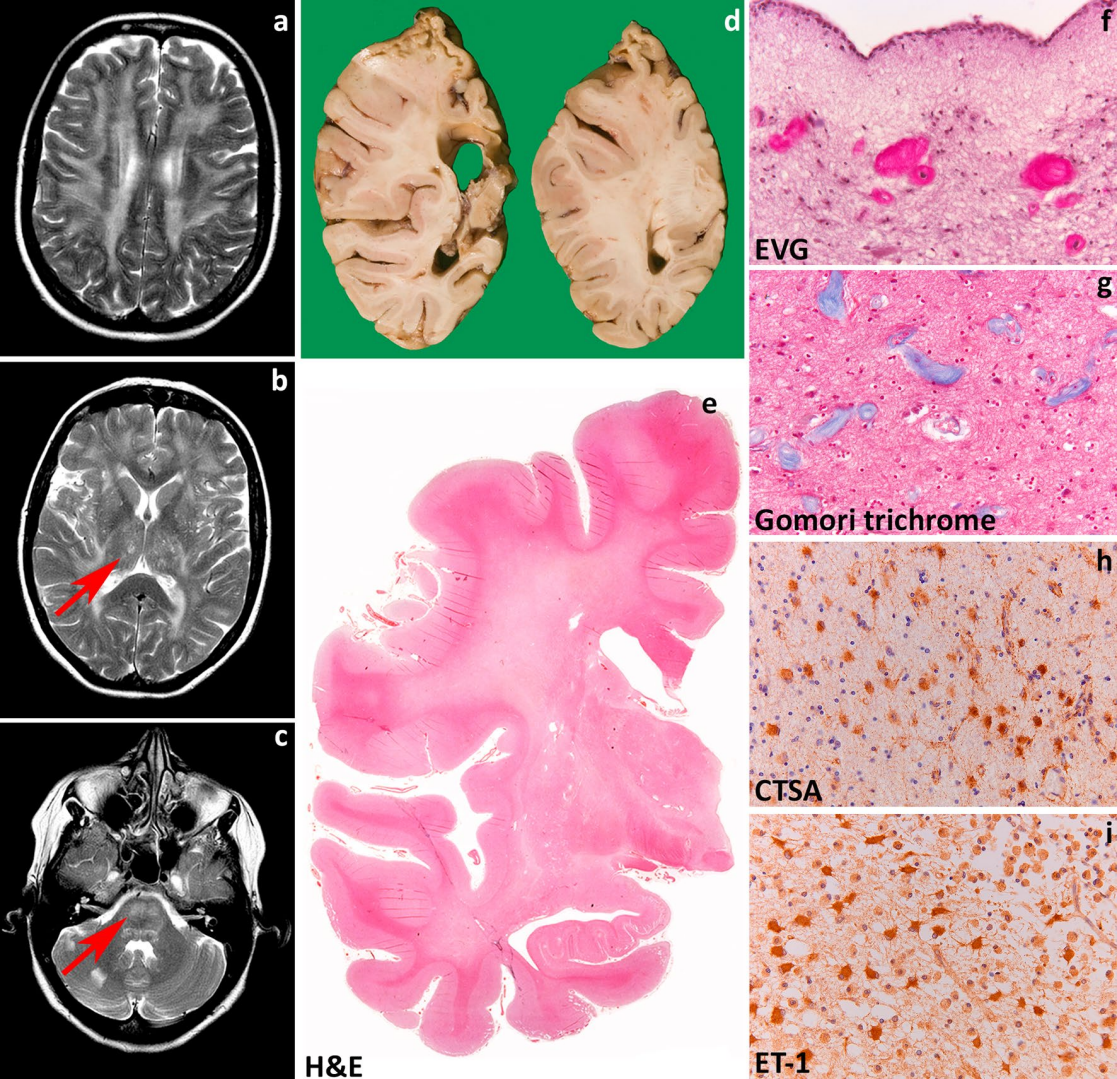
Cathepsin A-related arteriopathy with strokes and leukoencephalopathy. **a** T2-weighted axial image of a 52-year-old patient shows patchy and confluent signal hyperintensities in the periventricular and deep cerebral white matter. **b**, **c** T2-weighted axial image of the same patient show vascular lesions in the basal nuclei and thalami (**b**, *arrow*) and signal abnormalities in the basis of the pons (**c**, *arrow*). Two small infarctions in the right cerebellar hemisphere are visible (**c**). **d** Coronal sections of a cerebral hemisphere of 75-year-old patient show a cortico-subcortical infarct in the parietal lobe. The white matter appears relatively intact. **e** Whole mount of a coronal section of a 74-year-old patient stained with Haematoxylin & Eosin shows diffuse pallor of the periventricular and deep white matter with relative sparing of the subcortical areas and U-fibers. Note also small necrotic lesions in the periventricular white matter, basal nuclei and thalamus. **f** Elastic van Gieson stain of the periventricular white matter shows changes of the terminal arteriolar branches with asymmetric wall thickening and virtually complete lumen occlusion. **g** The same vascular changes are present in the deep white matter (Gomori trichrome). **h** Stain against cathepsin A (CTSA) shows strong immunoreactivity in white matter astrocytes. **i** In the same areas, astrocytes also robustly express endothelin-1 (ET-1)

Cathepsin A-related arteriopathy with strokes and leukoencephalopathy (CARASAL) is a novel hereditary adult-onset cerebral small vessel disease identified by whole exome sequencing in two presumably unrelated Caucasian Dutch families [31]. CARASAL patients share a dominant c.973C>T, p.(Arg325Cys) mutation in the *CTSA* gene encoding cathepsin A. Recessive *CTSA* mutations cause galactosialidosis, a rare systemic lysosomal storage disorder [34]. This opens to the possibility, not investigated so far, that heterozygous carriers of *CTSA* mutations causing galactosialidosis may also be at risk for small vessel disease. Patients with CARASAL present in their third to fifth decade of life with headaches or migraine, mild intellectual impairment and mild gait problems. Most develop overt signs of vascular disease with hypertension, transient ischemic attacks and strokes. Patients also have symptoms of brainstem dysfunction, including dry eyes and dry mouth with difficult swallowing. MRI shows T2-hyperintense signal changes in the frontal and parietal periventricular and deep white matter that are initially patchy and became confluent with time (Fig. 8a). The temporal poles are not affected. Multiple small signal abnormalities are additionally present in the basal nuclei, thalami, and brainstem (Fig. 8b, c). Infarcts, microbleeds and small hemorrhages may also be seen and are usually more prominent in older patients [31].

The neuropathology of three CARASAL patients has been reported [31]. On external examination, the brain is normal. Sectioning shows atrophy of the cerebral white matter and possibly scattered small infarcts in the white matter, deep cerebral gray structures, brainstem, and cerebellum (Fig. 8d, e). Microscopic examination reveals a diffuse involvement of the white matter with myelin pallor, reactive astrogliosis, normal to increased oligodendrocyte numbers and relative axonal preservation. Lacunar changes are present, although to a variable degree. Consistent with hypertension being an associated clinical sign, arterioles are diffusely sclerotic. CARASAL patients, however, show additional changes of the distal arteriolar branches throughout the cerebral white matter, basal nuclei and subependymal regions. These consist of remarkably asymmetric fibrous thickening of the vessel wall with loss of smooth muscle cells leading to near-complete occlusion of the lumen (Fig. 8f, g). These changes also involve the vasa vasorum.

The pathophysiology of CARASAL is still unclear. Cathepsin A stabilizes a lysosomal multi-enzyme complex with β-galactosidase and neuraminidase-1. Recessive *CTSA* mutations cause galactosialidosis due to deficiency of these enzymes [34]. In CARASAL, β-galactosidase and neuraminidase-1 activity are normal [31]. Considering the different phenotypes of galactosialidosis and CARASAL, a dominant-negative effect of the CARASAL mutation is not plausible. Alternatively, the CARASAL mutation may interfere with cathepsin A folding causing a neomorphic effect [76]. In line with this possibility, cathepsin A is overexpressed by CARASAL white matter astrocytes (Fig. 8h).

White matter lesions in small vessel diseases are thought to be ischemic in nature. Animal studies suggest that restriction of the vessel lumen leads to chronic white matter hypoperfusion resulting in repeated selective oligodendrocyte death and degeneration of myelinated fibers [162, 163, 170]. Other mechanisms could be involved, including blood–brain barrier damage [262], local subclinical inflammation [189], and oligodendrocyte apoptosis [27]. In the white matter of CARASAL patients, oligodendrocyte and OPC numbers are increased, possibly as a consequence of cathepsin A overexpression. Besides stabilizing β-galactosidase and neuraminidase-1, cathepsin A inactivates endothelin-1, a vasoactive peptide with roles in blood pressure regulation [105] and OPC maturation [82]. In multiple sclerosis, endothelin-1 overexpression by reactive astrocytes inhibits OPC maturation and remyelination [82]. Possibly due to reduced cathepsin A activity, the white matter of CARASAL patients harbors a higher abundance of astrocytic endothelin-1 than controls, including other small vessel diseases (Fig. 8i) [31]. Astrocyte-derived endothelin-1 may contribute to white matter damage by mediating persistent vasoconstriction and hypoxia and additionally by halting OPC maturation thus further impairing myelin maintenance and repair [31].

## Conclusions

The diagnostic approach combining MRI pattern recognition with next generation sequencing has remarkably increased the number of diagnosable genetic white matter disorders, and confirmed that many are due to defects in gene products specifically or also expressed in cell types other than the oligodendrocyte. The last decades have also witnessed a tremendous increase in the knowledge of the white matter demonstrating that all cell types inhabiting it are involved in its development, maintenance, function and repair. This has challenged the traditional myelin-centric view of leukodystrophies that is now proved surpassed. We support a novel definition of leukodystrophy that reflects the current knowledge: leukodystrophies are all genetically determined disorders primarily affecting the CNS white matter, irrespective of the structural white matter component involved, the molecular process affected and the disease course [103]. In the wake of this definition, we here propose a new classification of leukodystrophies based on a cellular pathology approach that takes into account the contribution of cell types other than oligodendrocytes and structures other than myelin driving white matter pathology, including astrocytes, axons, microglia and blood vessels. In reviewing the neuropathology and disease mechanisms of some leukodystrophies, we show that this classification also provides systematic additional information regarding the pathogenesis. The complicated interplay between the different white matter components in the healthy CNS necessarily implies that the diseases mechanisms underlying leukodystrophies are also complex. Our classification therefore also recognizes the possibility that a specific disease does not primarily affect only one cell type or structure and with that belongs to more than one category. Giant axonal neuropathy, for example, is due to defects in gigaxonin that maintains neuroaxonal cytoskeletal integrity and transport, but is also responsible for proper intermediate filament degradation in astrocytes. In other disorders, the neuropathology may be characterized by prominent secondary involvement of selected white matter components. MLC, for example, is due to a defective function of the astrocyte-specific protein MLC1, which is involved in astrocytic control of ion–water homeostasis. Although MLC is pathologically characterized by intramyelinic edema, this is a secondary phenomenon and MLC is thus categorized under the astrocytopathies. The implications of such information are important. We face a time in which new treatments are being explored, including cell-based replacement treatments, new drugs and small molecules, and therapies aiming at enhancing endogenous repair. It is conceivable that none of the leukodystrophies will be cured by a single treatment, but that they will rather benefit from a combined therapeutic approach. Knowledge on which cells types are involved in the pathophysiology of the single disorder and their disease mechanisms and interactions becomes thus crucial to develop a successful therapeutic strategy for these deleterious and often fatal diseases.
